# Synergistic Effects of Novel Xanthone Derivatives and Mild Hyperthermia in Ovarian Cancer: Insights from Gene Expression and In Silico Analyses

**DOI:** 10.3390/cancers17172896

**Published:** 2025-09-03

**Authors:** Jakub Rech, Dorota Żelaszczyk, Henryk Marona, Ilona Anna Bednarek

**Affiliations:** 1 Department of Biotechnology and Genetic Engineering, Faculty of Pharmaceutical Sciences in Sosnowiec, Medical University of Silesia, 40-055 Katowice, Poland; 2 Department of Bioorganic Chemistry, Chair of Organic Chemistry, Faculty of Pharmacy, Jagiellonian University Medical College, 30-688 Krakow, Poland; dorota.zelaszczyk@uj.edu.pl (D.Ż.);

**Keywords:** xanthones, synthetic xanthone derivatives, hyperthermia, chemotherapy, ovarian cancer, cellular stress, metastasis, HSP, gene ontology, gene set enrichment, molecular pathways

## Abstract

Background/Objectives: In the fight against ovarian cancer, various therapies have been employed, with a strong focus on developing novel derivatives of existing substances. Methods: In this study, we continue our research on novel xanthone derivatives in combination with mild hyperthermia, targeting ovarian cancer cell lines TOV-21G and SK-OV-3. Using qPCR arrays, we analyzed 84 cellular stress-related genes categorized into anti-oxidant and pro-oxidant enzymes, molecular chaperones, and xenobiotic metabolism including the cytochrome P450 group. Furthermore, we conducted in silico analyses to investigate the pathways of the most affected genes, gene set enrichment, and gene ontology. Results: The most significant changes were observed in SOD2, SOD3, CYP2F1, CYP1B1, and HMOX1. Additional changes related to drug toxicity and the postulated mechanism of action were also identified. Based on in silico analyses, we concluded that the primary node of hyperthermia-induced changes is HSPA1A. Heat-induced alterations predominantly revolve around misfolded proteins, monooxygenase activity, and ATPase activity. Conclusions: To summarize, the combined therapy of novel xanthone derivatives and mild hyperthermia shows promising results and warrants further investigation to fully elucidate the mechanisms of action underlying these effects.

## Simple Summary

Ovarian cancer treatments often focus on novel derivatives of existing substances. This study examines xanthone derivatives combined with mild hyperthermia on TOV-21G and SK-OV-3 ovarian cancer cell lines. We analyzed the expression of 84 stress-related genes, including anti-oxidant/pro-oxidant enzymes, molecular chaperones, and xenobiotic metabolism including the cytochrome P450 group. In silico analyses, gene pathways, enrichment, and ontology were explored. Significant changes were noted in genes expression which may potentially explain the mechanism of action of used substances. Additionally, altered genes may be involved in different cell death types. The combination of xanthone derivatives and mild hyperthermia demonstrates promising potential, meriting further investigation into the underlying mechanisms and potential further use as a novel anticancer therapy.

## 1. Introduction

Ovarian cancer is generally diagnosed in USA patients over 50 years (median 63 y.) with more than half of the cases diagnosed with distant malignancies [1,2]. Despite significant progress in oncology treatment, it still remains a significant challenge with 50% having a 5-year survival rate [1]. Current standard treatments primarily involve chemotherapy and surgical intervention, but their effectiveness is limited, driving the search for new therapeutic strategies.

Emerging therapies focus on enhancing drug delivery, combining therapeutic agents, and developing new compounds based on well-established drugs and delivery systems used in cancer and other diseases. The use of various delivery vehicles holds great promise, potentially offering prolonged drug release, extended cell exposure, and reduced drug concentration requirements [3,4,5,6,7]. Another concept involves utilization of an additional stimulating agent such as temperature elevation. The use of hyperthermia against cancer has a long history of use, despite the lack of precise mechanism for an action description. Heat may increase cell membrane permeation and increase drugs efficiency but also stimulate the expression of stress-related proteins which promote cell survival, including cancer cells. In this study we wanted to investigate whether the mutual stimulation of drugs with hyperthermia will decline HSP expression and increase the death rate of cancer cells.

Currently, majority of research are focused on drug repurposing or validating novel derivatives of known substances to improve their anticancer properties. This approach aims to improve therapeutic efficacy by introducing small structural modifications to compounds with known mechanisms of action and side effects. These modifications can be derived from natural products such as herbs and fruits or by altering synthetic agents. In addition to enhancing therapeutic effects, this strategy may also lead to the creation of unique substances capable of overcoming drug resistance in cancer cells. α-Mangostin and gambogic acid are examples of substances of natural origin, with promising anticancer functions toward different cancer types [8]. In our previous articles, we described the influence of synthetically obtained xanthones (compounds **C7** and **C8**, Table 1) on the ovarian cancer cells on the cellular and molecular level [9,10] including induction of ovarian cancer cell apoptosis, decreased wound-healing efficiency and clonogenic potential, and changes in mitochondria membrane potential and total mass. Also, the xanthones increased the expression of heat shock protein 90A, 90B, and Hsc70. Additionally we initially screened the influence of different hyperthermia temperatures on tested cells and chose 39 °C as appropriate for this study [9]. A summary of the GI10 (concentration of selected drug that inhibits cell growth by 10%) of compounds utilized in the study is provided in Table 1. An additional stimulant of simultaneous mild hyperthermia treatment was also utilized to further improve the anticancer effect of tested substances.

Among many functional proteins in cells, stress-related ones are eagerly researched to unveil their role in the dysregulation of cell cycle and cancer progression. These proteins are responsible for appropriate response of various stressors and changes in cell environment. Thus, knowledge about their dysregulation in cancer cells, especially in response to anticancer therapy, is important as it may administer drug resistance and cell survival [11,12].

In this study, we continue our research on two aminopropyl morpholine xanthone derivatives (**C7** and **C8**), evaluating their biological activity in two ovarian cancer cell lines with distinct characteristics. TOV-21G cells originate from ovarian clear cell adenocarcinoma (primary tumor origin), while SK-OV-3 cells come from ovarian serous cystadenocarcinoma (metastatic tumor origin). Our qPCR arrays focused on stress-related genes that revealed genes engaged in response to hyperthermia, novel xanthone derivatives, and control drugs. We conducted in silico studies to further investigate the main proteins responsible for response to hyperthermia and drugs used.

## 2. Results and Discussion

### 2.1. qPCR of Cellular Stress-Related Proteins

qPCR method is commonly used to estimate the changes in gene expression stimulated by different conditions and xenobiotics. In this study we used hyperthermia combined with the treatment of four different compounds. Two of them were xanthone derivatives, previously selected for their influence on cells behavior, and the other two were α-mangostin (**MAG**) and cisplatin (**CIS**), as reference compounds to known xanthone and a commonly used chemotherapy drug, respectively.

In our study we focused on stress-related proteins, as their influence on cells contributes to cancer malignancy and drug resistance. qPCR arrays covering 84 genes were used to efficiently investigate many genes in one assay. These genes are grouped and listed in Table 2. For analysis we choose genes with expression regulation fold exceeding 2/−2 as significant. Untreated 37 °C cells were used as control for all qPCR analysis.

#### 2.1.1. Anti-Oxidant and Pro-Oxidant Enzymes

**Figure 1:**
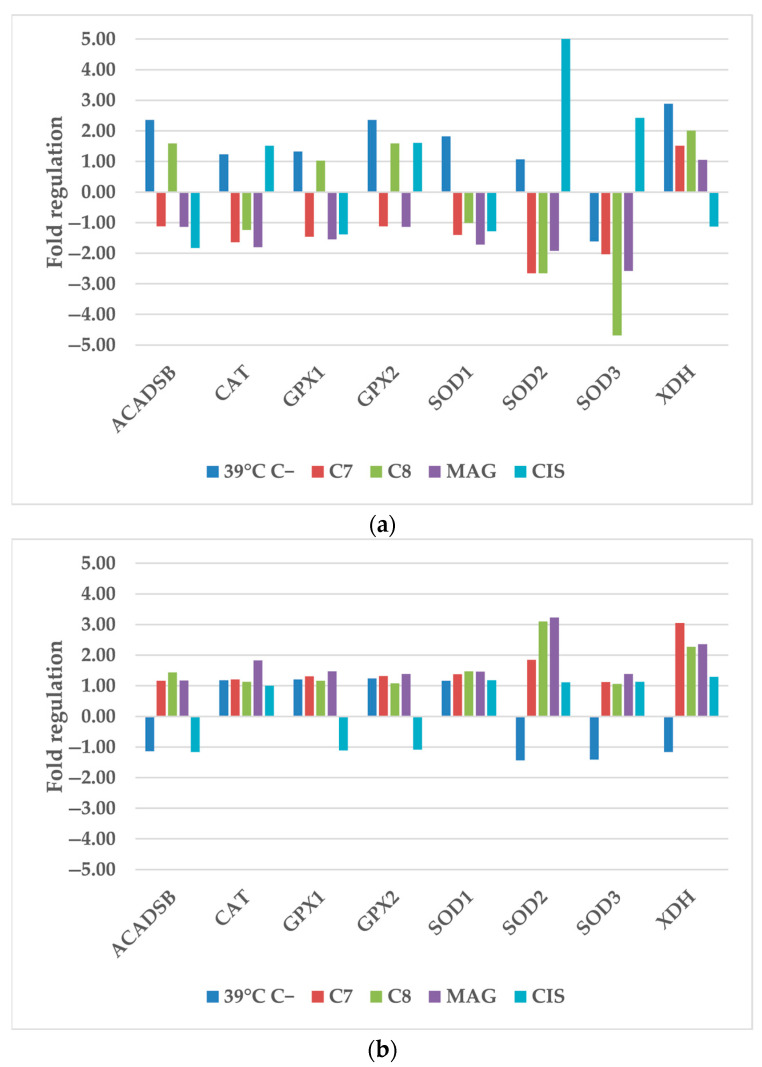
The regulation fold of anti-oxidant and pro-oxidant enzymes of (**a**) TOV-21G and (**b**) SK-OV-3 cells after treatment of hyperthermia and selected compounds. Gene expression values were cropped to a range of −5 to 5 to ensure appropriate data presentation. Excessing TOV-21G SOD2 fold regulation: **CIS**−5.42. 39 °C C−—cells treated only by 39 °C; **C7**—compound 7; **C8**—compound 8; **MAG**—α-mangostin; **CIS**—cisplatin.

The increment of Reactive Oxygen Species (ROS) is the postulated mode of action of xanthones and our studied derivatives. In this study, out of eight tested ROS-related genes, five of them were dysregulated. These genes are ACADSB (acyl-CoA dehydrogenase short/branched chain), GPX2 (glutathione peroxidase 2), SOD2 (superoxide dismutase 2), SOD3 (superoxide dismutase 3), and XDH (xanthine dehydrogenase) (Figure 1). There is also clear difference between cell lines, where the response of SK-OV-3 cells was much lower, and only SOD 2 and XDH genes were dysregulated, where the rest of them remained almost unchanged.

The response of TOV-21G cells were much different from SK-OV-3 cells, and also depended on the use of xenobiotic. Hyperthermia alone upregulated all TOV-21G genes, despite SOD3. Only ACADSB, GPX2 and XDH were upregulated by more than 2-fold. Hyperthermia combined with **CIS** increased SOD2 and SOD3 expressions to 5.42 and 2.43, respectively. Other used compounds decreased SOD2 and SOD3 expression. **C7** and **C8** had most impact on SOD2; while on SOD3, **MAG** also decreased the expression more than 2-fold.

Different results were obtained from SK-OV-3 cells. Previous results indicated that SK-OV-3 cells are more vulnerable to increased temperature than TOV-21G cells [9]. In these results, hyperthermia alone had almost no impact on gene expression changes. **C8** and **MAG** combined with hyperthermia both increased the SOD2 expression over 3-fold. XDH gene expression was upregulated by **C7**, **C8**, and **MAG**.

Our previous study, investigating only selected compounds treatment without hyperthermia, presented similar results [10]. The most influenced TOV-21G genes were also SOD2, SOD3, and XDH, and for SK-OV-3 were SOD2 and XDH. Additional hyperthermia treatment of TOV-21G, has boosted **C7** and **CIS** activity on SOD2. Most significant changes in additional hyperthermia stimulus were observed in the **CIS** treatment of SOD2. Its expression elevated to over 5 from initial repression by **C8** alone. Simultaneous hyperthermia and **C7** treatment were less influential on SOD3 expression, but **C8** with additional hyperthermia was superior to **C8** alone. XDH expression did not change drastically compared to drugs alone, but additional heat stimulus reversed the **CIS**-enhancing influence to suppression.

These results present cell line-specific response to hyperthermia and xenobiotics, supporting previously published results [13]. The inhibition of dismutases is the expected mode of action of the compounds used in this study [10]. These compounds are also effective enough to breakout of hyperthermia ROS-inducing activity. The structure of derivatives is an important factor, determining cellular ROS-level induction or the scavenging properties of compound [14,15].

These findings align with previous studies on other xanthones, such as Cluvenone, which has been shown to modulate gene expression by activating stress-related pathways, including the MAPK and Nrf2 signaling cascades. Cluvenone also induces intracellular ROS production, further supporting the idea that certain xanthones can influence cellular transcriptional responses [16]. This suggests that the gene-regulatory potential observed in our study may be a common feature among bioactive xanthone derivatives.

**CIS** seems to cooperate with heat and, additionally, boosts ROS levels in cancer cells, which is visible in the significant stimulation of SODs expression. This hypothesis should be additionally validated through research but current studies are in line with it [17,18,19].

#### 2.1.2. Xenobiotic Metabolism—P450

Xenobiotic metabolism genes are one of the key components of the cellular system responsible for drug metabolism and resistance mechanisms. Current research are focused on overcoming multi-drug resistance and restoring cells sensitivity. Cytochrome P450 isoforms are involved in a wide range of biochemical reactions, and are cardinal for appropriate endo- and exo-genous compound metabolism.

**Figure 2:**
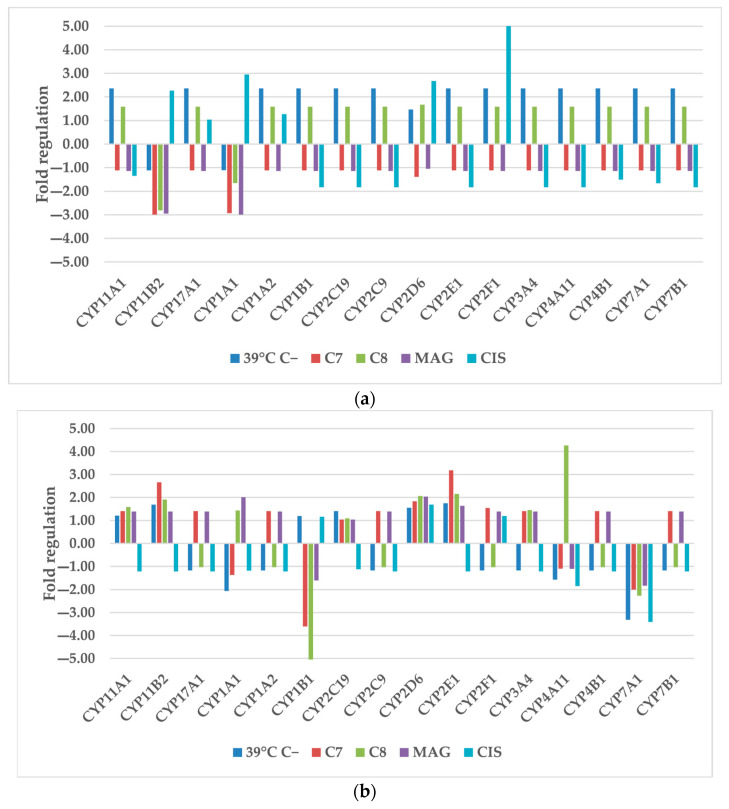
The regulation fold of xenobiotic metabolism—P450 isophorms of (**a**) TOV-21G and (**b**) SK-OV-3 cells after treatment of hyperthermia and selected compounds. Gene expression values were cropped to a range of −5 to 5 to ensure appropriate data presentation. Excessing TOV-21G CYP2F1 fold regulation: **CIS** 5.10; SK-OV-3: CYP1B1 **C8** −7.57. 39 °C C−—cells treated only by 39 °C; **C7**—compound 7; **C8**—compound 8; **MAG**—α-mangostin; **CIS**—cisplatin.

TOV-21G cells exposed to hyperthermia alone increased the expression of almost all investigated P450 isoforms (Figure 2). Additional xenobiotic stimulus seemed to reduce these changes. Only drugs-treated CYP11B2 (cytochrome P450 family 11 subfamily B member 2), CYP1A1 (cytochrome P450 family 1 subfamily A member 1), and **CIS**-treated CYP2D6 (cytochrome P450 family 2 subfamily D member 6) and CYP2F1 (cytochrome P450 family 2 subfamily F member 1) expression have changed in significant manner. All mentioned genes expression were elevated by **CIS** treatment with CYP2F1 having most elevation (5.10). **C7**, **C8** and **MAG** lessened the fold expression of CYP11B2, CYP1A1 in the range of −1.65 to −2.99. Worth mentioning is also the consequent increment to over 1.50 fold of all genes except CYP11B2, CYP1A1 by **C8** treatment.

Also, the fold expression of SK-OV-3 CYP did not change significantly by hyperthermia stimulus alone. Only CYP1A1 and CYP7A1 (cytochrome P450 family 7 subfamily A member 1) were cut down to −2.06 and −3.32, respectively. Major changes were observed for CYP11B2 after **C7** treatment, CYP1A1 after **MAG** treatment, CYP1B1 (cytochrome P450 family 1 subfamily B member 1) after **C7** and **C8** treatment, CYP2D6 after **C8** and **MAG** treatment, CYP2E1 (cytochrome P450 family 2 subfamily E member 1) after **C7** and **C8** treatment, CYP4A11 (cytochrome P450 family 4 subfamily A member 11) after **C8** treatment, CYP7A1 after **C7**, **C8** and **CIS** treatment.

CYP1A1 contributes to the transformation of xenobiotics with pro-carcinogenic properties into active forms, and its downregulation may be associated with slower cancer progression. Thus elevated expression by **MAG** is not promising. Such phenomena was not observed for the drug alone, so we conclude it is connected with simultaneous hyperthermia treatment [10]. It also seems that inhibiting activity of drugs to cytochrome isoforms is cell specific (TOV-21G CYP1A1; SK-OV-3 CYP1B1). In contrast, CYP1B1 is overexpressed in the majority of ovarian cancers, with no such elevation observed in normal tissues [20]. Aryl hydrocarbon receptor (AhR) is stimulated by xenobiotics, CYP1A1 and CYP1B1, leading to the conversion of procarcinogens into carcinogens. In ovarian cancers, this pathway activates the PI3K/AKT signaling cascade, promoting a mesenchymal–epithelial transition and contributing to metastasis [21].

CYP2D6 activity is described to regulate the effectiveness of tamoxifen, a drug primarily used in breast cancer therapy but also utilized in ovarian tumors that does not respond to previous chemotherapy [22]. Dysregulation of this gene may also be a factor corresponding to cell response to the drugs.

CYP11B2 is involved in aldosterone synthesis in adrenal glands, and it is postulated that its elevated expression is an ongoing hormone synthesis that is correlated with cell proliferation by GnRHR/LHRH (gonadotropin-releasing hormone receptor; luteinizing hormone-releasing hormone) [23]. Studies on ovarian cells describe an anti-apoptotic effect while GnRHR/LHRH receptors were stimulated, which resulted in an NFκB activation and apoptosis reduction [24]. Decreased expression of CYP11B2 may additionally stimulate pro-apoptotic changes in ovarian cancer cells.

CYP2F1 is a hydroxylase with monooxygenase and oxidoreductase activity, primarily expressed in the lungs for various pulmonary toxins removal. It was also detected in significantly higher levels in ovarian cancer compared to a normal ovary, thus it was proposed to serve as a prognostic marker [25]. Due to its function, overexpression may be a result of simultaneous heat and **CIS** treatment, but no other supporting data was discovered. Additionally, previous research indicate that **CIS** treatment alone inhibits this isoform expression [10].

CYP2E1 expression corresponds to proinflammatory IL-6, IL-8, and TNF-α proteins upregulation. Previously, the response to drugs alone was much stronger (specifically for SK-OV-3), and hyperthermia seems to dilute this effect [10,26].

CYP7A1 (cholesterol 7 alpha-hydroxylase) is another candidate for the ovarian cancer prognostic marker. It is a bile acids synthase, first-in-order, and rate-limiting enzyme of the bile acids synthesis pathway [27]. The combined therapy seems to have a smaller influence on cells compared to the changes induced by drugs alone [10]. In the liver, CYP7A1 expression is restricted in proinflammatory conditions to reduce the acids synthesis. Excess cholesterol is also metabolized to precursors for steroid hormones by CYP11A1, mostly in steroidogenic tissues.

Elevated CYP4A11 levels in serum were found in patients with nonalcoholic fatty liver disease. Also, the increased levels of cytochrome correlated with high levels of lipid peroxidation products. These were produced in response to high ROS levels in the liver [28]. This is in line with postulated ROS-induced mechanism of action of our xanthone derivatives. No CYP4A11 on ovarian cells was found after data investigation.

#### 2.1.3. Other Xenobiotic Metabolism Genes

**Figure 3:**
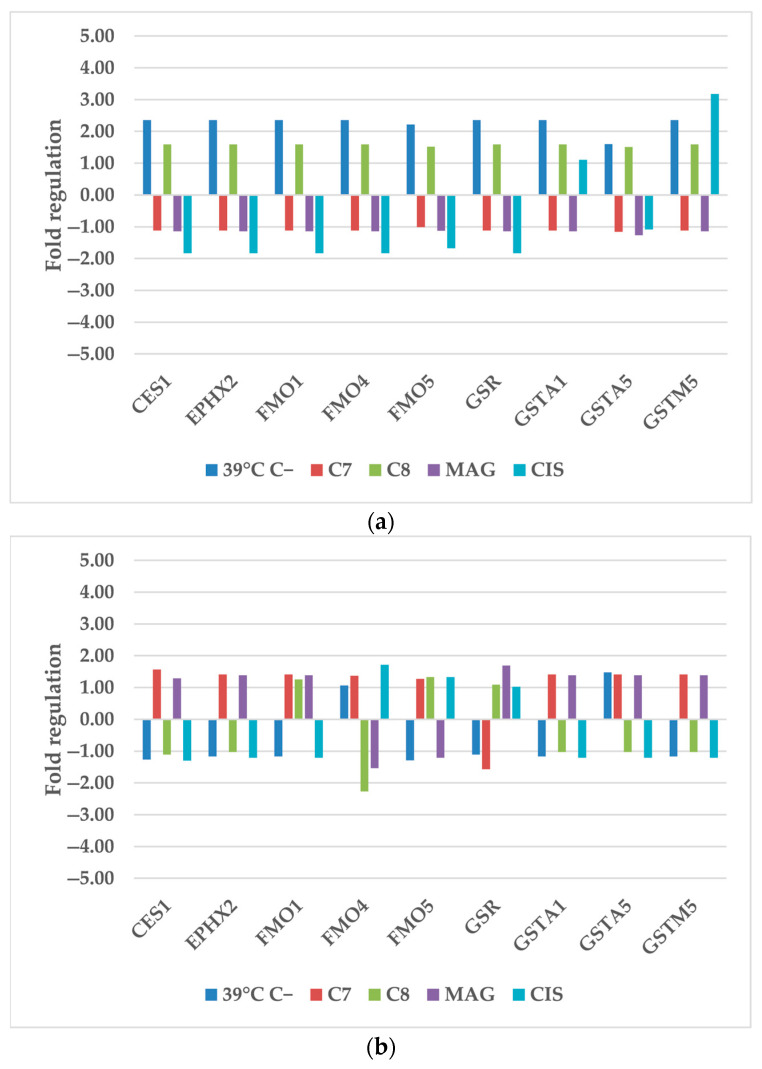
The regulation fold of other xenobiotic metabolism genes of (**a**) TOV-21G and (**b**) SK-OV-3 cells after treatment of hyperthermia and selected compounds. 39 °C C−—cells treated only by 39 °C; **C7**—compound 7; **C8**—compound 8; **MAG**—α-mangostin; **CIS**—cisplatin.

Hyperthermia promoted the expression of all other xenobiotic metabolism genes of TOV-21G, besides GSTA5 (glutathione S-transferase alpha 5) (Figure 3). Previous TOV-21G qPCR showed a similar trend [10]. Only GSTM5 (glutathione S-transferase mu 5) expression was elevated significantly by **CIS** treatment. GSTM5 belongs to glutathione S-transferases (GSTA) enzymes. These enzymes are responsible for ROS removal and also the detoxification of various xenobiotics by coupling them with glutathione. Moreover the whole GSTM family is connected with cancer progression, patient survival, and chemoresistance to drugs [29]. Previously, the cells gene expression response to **C7** and **C8** was much stronger [10]. Additional heating seemed to normalize the changes. Hyperthermia is a stronger gene-expression-dysregulation agent than its combination with drugs.

SK-OV-3 expression was varied only for FMO4 (Dimethylaniline monooxygenase 4) treated by heat and **C8** but in a lower manner than **C8** alone [10]. This enzyme is also involved in ROS removal and detoxification of drug nucleophilic heteroatom centers. It might also have its role in drug resistance [30,31].

#### 2.1.4. Heat Shock Proteins

Heat shock proteins are the majority among the investigated genes. They comprise a highly conservative gene family, assisting in protein folding, preventing aggregation, protein refolding or degradation of misfolded one. HSP genes levels are upregulated in response to various agents, such as drugs or hyperthermia, being most important one. Their natural function is to maintain homeostasis and support cell survival, but in cancer cells they can often promote proliferation and inhibit drug susceptibility.

**Figure 4:**
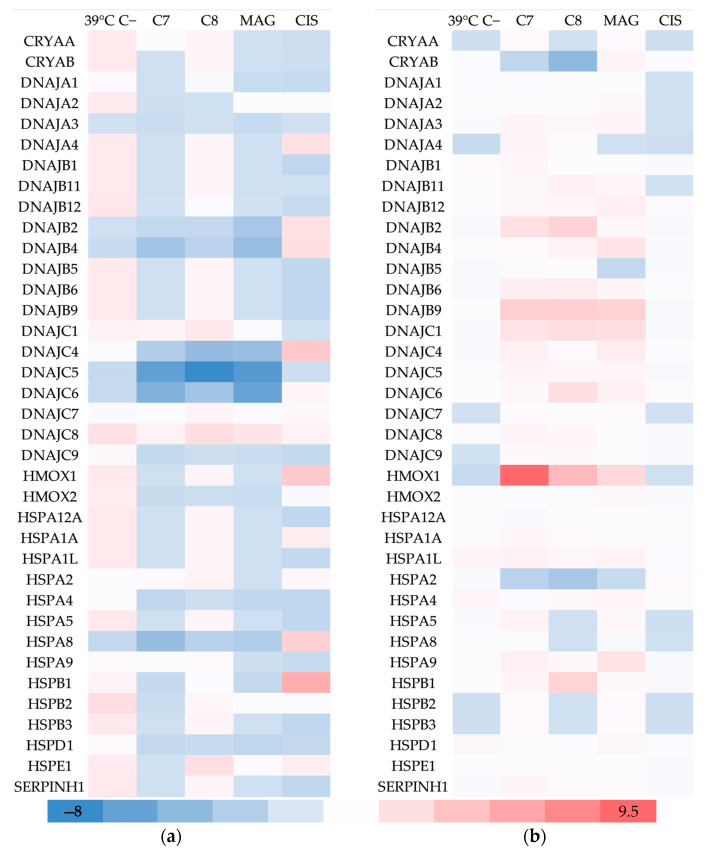
The regulation fold of heat shock proteins of (**a**) TOV-21G and (**b**) SK-OV-3 cells after treatment of hyperthermia and selected compounds. 39 °C C−—cells treated only by 39 °C; **C7**—compound 7; **C8**—compound 8; **MAG**—α-mangostin; **CIS**—cisplatin.

Hyperthermia elevated almost all investigated TOV-21G genes except for DNAJA1, DNAJA2, DNAJB2, DNAJB4, DNAJC1-**C7**, DNAJC9, HSPA2, HSPA4, HSPA8, HSPA9, HSPB1, and HSPD1 (Figure 4). Additionally, among multiple genes, a pattern can be seen, where hyperthermia and **C7** both increase the expression even if it is not significant. Other genes influenced by xenobiotics were: DNAJA4 (DnaJ heat shock protein family (Hsp40) member A4) (**CIS**), DNAJB2 (DnaJ heat shock protein family member B2) (**MAG**, **CIS**), DNAJB4 (DnaJ heat shock protein family member B4) (**C7**, **MAG**, **CIS**), DNAJC1 (DnaJ heat shock protein family member C1) (**C8**), DNAJC4 (DnaJ heat shock protein family member C4) (**C7**, **C8**, **MAG**, **CIS**), DNAJC5 (DnaJ heat shock protein family member C5) (**C7**, **C8**, **MAG**), DNAJC6 (DnaJ heat shock protein family member C6) (**C7**, **C8**, **MAG**), DNAJ**C8** (DnaJ heat shock protein family member **C8**) (**C8**, **MAG**), HMOX1 (heme oxygenase 1) (**CIS**), HSPA1A (heat shock protein family A (Hsp70) member 1A) (**CIS**), HSPA8 (heat shock protein family A (Hsp70) member 8) (**C7**, **C8**, **MAG**, **CIS**), HSPB1 (heat shock protein family B (small) member 1) (**CIS**), and HSPE1 (heat shock protein family E (Hsp10) member 1) (**C7**, **CIS**).

The mainly influenced SK-OV-3 genes were CRYAB (crystallin alpha B) (**C7**, **C8**), DNAJB2 (**C7**, **C8**) DNAJB4 (**MAG**), DNAJB5 (DnaJ heat shock protein family member 5) (**MAG**), DNAJB6 (DnaJ heat shock protein family member 6) (**C7**, **C8**), DNAJB9 (DnaJ heat shock protein family member 9) (**C7**, **C8**, **MAG**), DNAJC1 (**C7**, **C8**, **MAG**), DNAJC4 (**C7**, **MAG**), DNAJC6 (**C7**), HMOX1 (**C7**, **C8**, **MAG**), HSPA2 (heat shock protein family A (Hsp70) member 2) (**C7**, **C8**), HSPA9 (heat shock protein family A (Hsp70) member 9) (**MAG**), and HSPB1 (**C8**).

Hyperthermia-influenced CRYAA (crystallin alpha A) and CRYAB genes are both part of the Hsp20 Small Heat Shock Proteins family. Elevated temperature may disrupt normal protein folding and their native structure, leading to lower solubility and loss of function. The Hsp20 proteins inhibit these unwanted effects by creating large soluble aggregates of denatured proteins. Stronger presence of these genes increases cell survival and suppresses apoptosis, both in normal and cancer cells. In the case of ovarian cancer, elevated levels of CRYAB are recognized as bad prognosis factors. They were found particularly in patients with poor outcomes and short recurrence-free survival. Additionally, TRAIL and **CIS** induced apoptosis was attenuated in ovarian cancer [32].

DNAJA4, DNAJB2, DNAJB4, DNAJB5, DNAJB6, DNAJB9, DNAJC1, DNAJC4, DNAJC5, DNAJC6, DNAJC8 are all members of the Hsp40 family. Their main function is helping chaperones, regulating the function of the main HSP70 and HSP90 proteins. Plenty of HSP40 genes are necessary for specific or more general substrate recognition and activation of HSP70 [33]. Final outcome of these proteins can vary drastically from pro- to anticancerous, depending on the cell in regard [34].

HSPA2, HSPA8, HSPA9 are members of the HSP70 family. As previously mentioned, their expression is related to the proteins activity of Hsp40. HSPA2 and HSPA9 were found to be under- and overexpressed, respectively, at gene and protein level in various cancer types, like ovarian, endometrial, breast, and lung [35]. HSPA8 is being connected with **CIS** resistance in ovarian cancer cells [36]. This protein seems to promote the degradation of caseinolytic protease P (CLPP) mediating in autophagy. Overexpression of CLPP increases apoptosis and ROS levels, and high levels of HSPA8 reverses this, promoting cancer progression. In this study only TOV-21G responded in this manner to therapy.

HSPB1 overexpression is correlated with cancer progression. This protein belongs to the Small Heat Shock Protein family (sHSP), and is postulated to be both a marker and indicator of ovarian cancer. Additionally it was found to be involved in many other cancer types such like breast, endometrial, and prostate [37].

HSPE1 was also found taking part in ovarian cancer development. Its hyperactivity can inhibit the immune response of T-cells. Its high expression may be a cellular response to drugs exposure, similar to previous research [37,38].

SK-OV-3, after stimulation by **C7**, **C8** and **MAG**, overexpressed HMOX1 (heme oxygenase 1 gene). In connection to SOD2 upregulation by the same agents, this may indicate ferroptosis, an iron-dependent type of cellular death, distinguished from apoptosis [39]. This hypothesis needs further confirmation, as no elevation was observed to GPX1–4 (glutathione peroxidase 1–4) genes. Such phenomena are not visible in TOV-21G cells, only **CIS** induced significant changes in expression of HMOX1 and SOD1 (superoxide dismutase 1), but no other mentioned ferroptosis-connected genes.

Out of tested compounds, **C7** is most potent in dysregulating the HSP genes. Its mode of action should be further investigated to unveil its full potential.

#### 2.1.5. Other Molecular Chaperones

**Figure 5:**
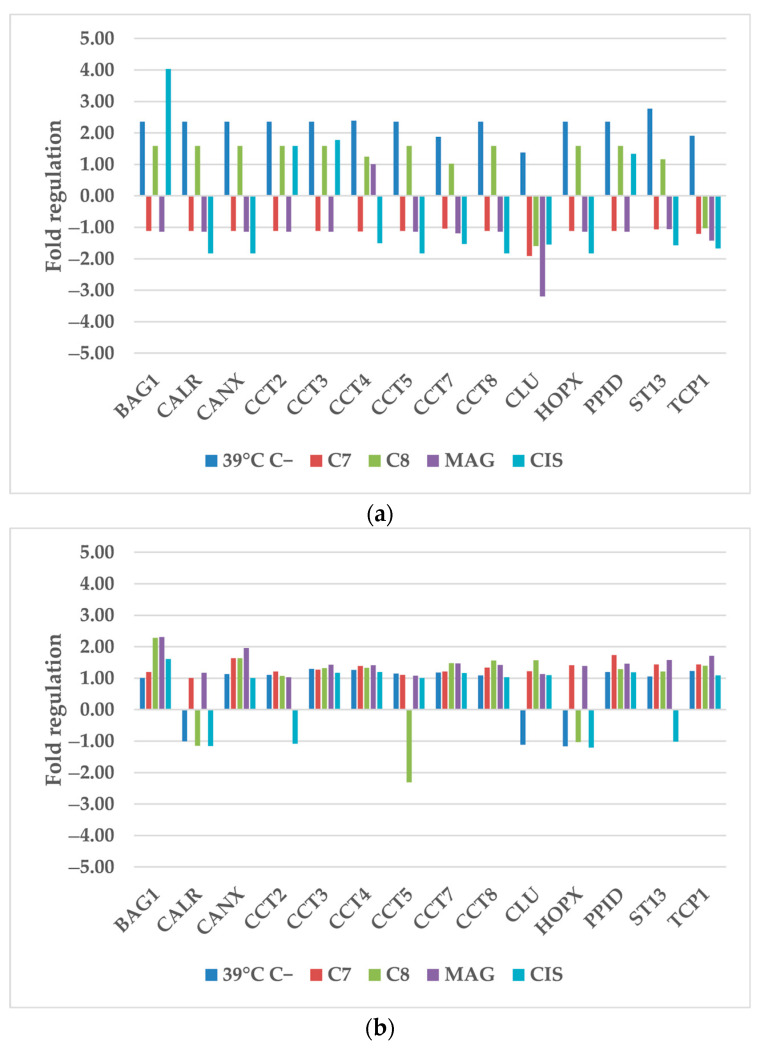
The regulation fold of other molecular chaperones of (**a**) TOV-21G and (**b**) SK-OV-3 cells after treatment of hyperthermia and selected compounds. Gene expression values were cropped to a range of −5 to 5 to ensure appropriate data presentation. 39 °C C−—cells treated only by 39 °C; **C7**—compound 7; **C8**—compound 8; **MAG**—α-mangostin; **CIS**—cisplatin.

TOV-21G under hyperthermia exposure, presented an increase in all other molecular chaperones expression besides CCT7 (chaperonin containing TCP1 subunit 7), CLU (clusterin), and TCP1 (t-complex 1) (Figure 5). Other genes expression were elevated over 2- times fold. Additional xenobiotics treatment mostly negated hyperthermia changes, except for BAG1 (Bcl-2-associated athanogene) after **CIS** exposure, and CLU after **MAG** treatment.

CLU gene function depends on whether it is present in nuclear or in free, secreted isoform. Suppressed expression of CLU requests for higher cancer death, and lower progression [40,41].

SK-OV-3 cells were resistant to hyperthermia, and further addition of xenobiotics influenced only BAG1 (**C8**, **MAG**) and CCT5 (T-complex protein 1 subunit epsilon) (**C8**).

BAG1 protein was found to be highly expressed in leukemia and its suppression activated proapoptotic pathways, vastly increasing cancer death [42]. It regulates cell apoptosis by inducing Bcl-2 by HSP70, by chaperoning HSP70. BAG proteins are also activated under stress conditions, increasing cell survival.

CCT5 encodes protein as being part of the TRiC (T-complex protein Ring Complex) complex. This complex role is to assist in the folding of various proteins in an ADP dependent manner.

Interestingly the changes induced by the drug alone were distant from these induced by simultaneous drug and hyperthermia treatment. This fact may be correlated by prominent changes in expression induced by hyperthermia alone. This factor influenced majority of investigated genes, thus its impact on cells cannot be omitted while comparing the drugs + hyperthermia vs. drugs alone activity. As most of drugs have at least partial specificity and target receptors/proteins, hyperthermia acts on the cell as a whole. Hyperthermia was designed to be an additional supporting factor of drug treatment, but due to its general mode-of-action it is hard to predict the direct changes on drugs tested. The genes- expression-dysregulation pattern is different from the drugs-alone pattern.

### 2.2. In Silico Analysis

#### 2.2.1. Gene Set Enrichment Analysis

To further evaluate the obtained data, the gene set enrichment analysis and gene ontology was provided, using the FunRich program v3.1.4 [43]. Hyperthermia and each drug + hyperthermia treatment was investigated separately. Only the genes with changes in expression exceeding 2/−2 were carried out in this section.

**Figure 6:**
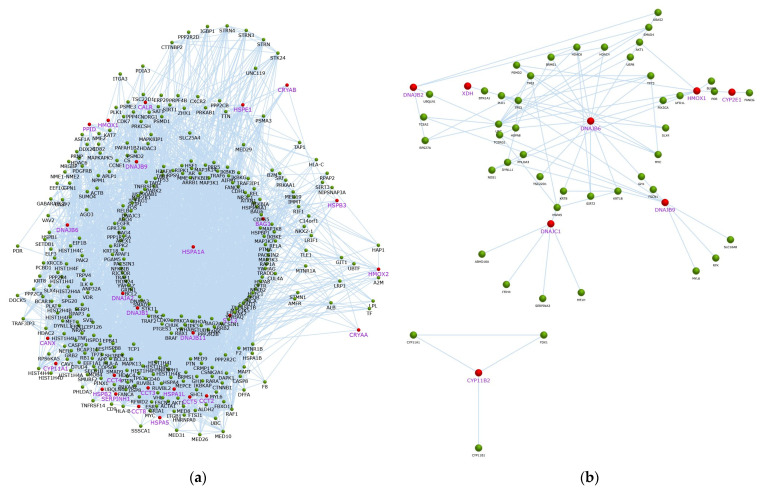
The network of interaction of overexpressed genes of (**a**) TOV-21G and (**b**) SK-OV-3 after heat treatment. Red nodes with purple text are overexpressed genes from our study, green nodes are genes out of our gene pool but found to have interaction with other genes.

Validation of investigated genes indicated HSPA1A as the main node (Figure 6). This node was also preserved when other, not investigated genes were also included. Interactions of other deregulated genes can be found in Supplementary Material.

**Figure 7:**
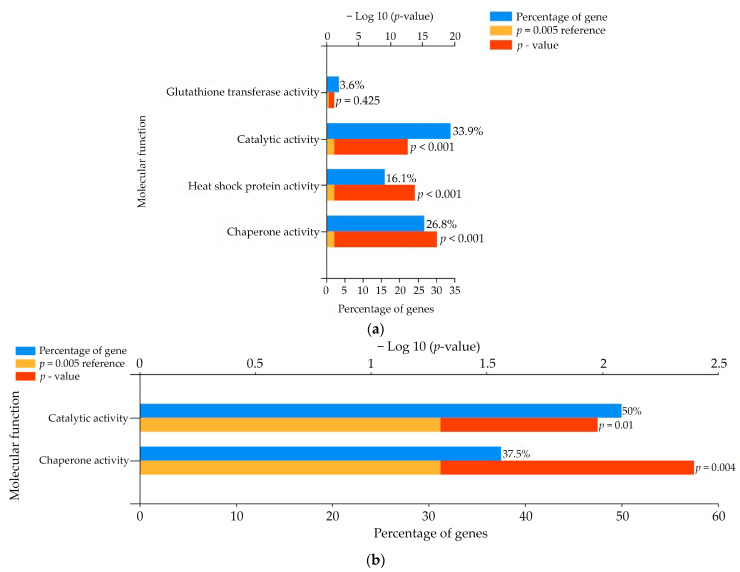
The molecular function of upregulated genes by hyperthermia of (**a**) TOV-21G and (**b**) SK-OV-3. Only genes with fold change below −2 or above 2 undergo the analysis.

Heat can alter the native structure of proteins by its disruption and misfolding. The gene ontology (GO) of hyperthermia alone treated TOV-21G indicates altered molecular functions of cells by means of misfolding the proteins and induction of chaperones binding to them for repair (Figure 7). Monooxygenases activity and heme binding changes are also expected, and were revealed in our study. Another interesting fact is the change in ATPases activity what can influence a lot of biological processes that are ATP-dependent. Majority of genes may be found in endoplasmic reticulum, microsomes, and exosomes. Investigation of exosomes and their influence on other cancer cells and microenvironment is our next goal. Molecular function of genes deregulated by drugs used, mostly revolves around HSP activity, chaperone activity, and superoxide dismutase activity (Supplementary Material).

#### 2.2.2. Pathway Analysis

**Figure 8:**
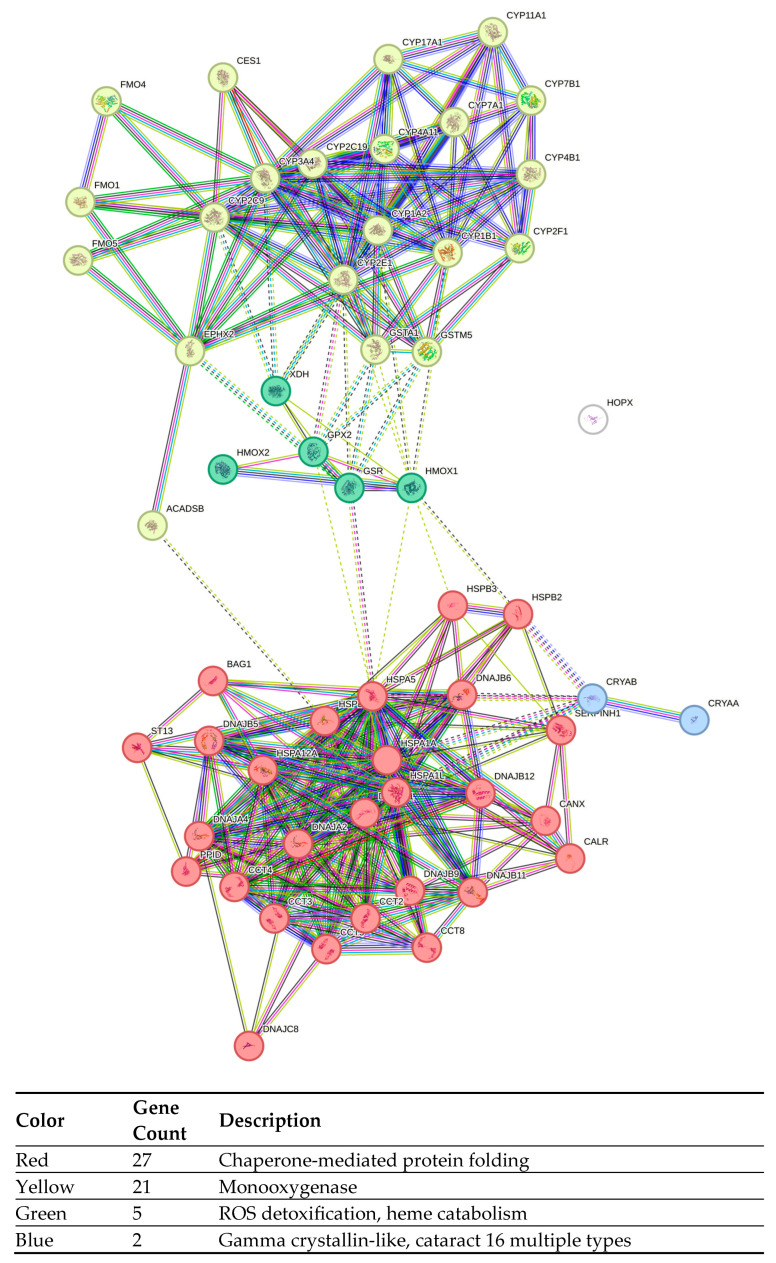
STRING proteins interaction pathway analysis of hyperthermia-treated TOV-21G proteins were grouped into clusters, based on their molecular function. Dotted lines represent the edges between clusters.

**Figure 9:**
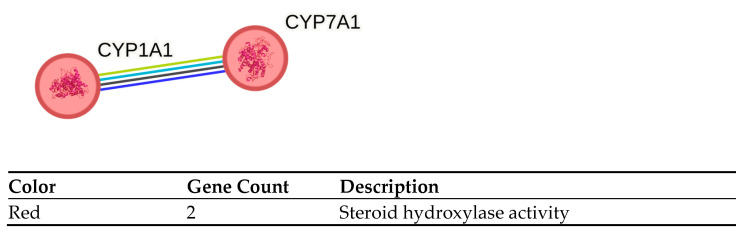
STRING proteins interaction pathway analysis of hyperthermia-treated SK-OV-3 cells. Proteins were grouped into clusters, based on their molecular function. Dotted lines represent the edges between clusters.

To further investigate the properties of drugs used and hyperthermia, the pathway analysis was performed using STRING v12.0 database. All genes significantly changed for each condition (drug or drug + hyperthermia) were loaded into the database, irrespective of up or downregulation. Genes were grouped in MCL natural clusters respective to their major molecular function (Figure 8 and Figure 9). Due to the restricted set of genes analyzed, the majority of clusters obtained were focused on chaperones, detoxification and removal of ROS, and SOD family activity. Full pathway analysis can be found in Supplementary Material.

## 3. Materials and Methods

### 3.1. Xanthone Derivatives Synthesis and Preparation

The synthesis and purification methods for aminopropyl morpholine xanthone derivatives, along with their physicochemical properties, were previously detailed in our earlier publication [9]. Both compounds (**C7**, **C8**) underwent analysis using ^1^H and ^13^C nuclear magnetic resonance (NMR) and mass spectrometry. Additionally, the melting points of all compounds were measured [9].

Stock solutions of xanthones were prepared by dissolving them in DMSO at a concentration of 10 mM each, and then stored at −20 °C. Just before the assays, the stock solutions were thawed and diluted to the desired concentration in DMEM.

### 3.2. Cell Culture and Treatment Conditions

In this study, two ovarian cancer cell lines, TOV-21G (ATCC^®^ CRL-11730™) and SK-OV-3 (ATCC^®^ HTB-77™), were utilized. Both cell lines were cultured in DMEM High Glucose with L-glutamine (Gibco, ThermoFisher Scientific, Dublin, Ireleand), supplemented with 10% FBS (PAN Biotech, Aidenbachch, Germany) and 1% PenStrep (Gibco, ThermoFisher Scientific, Dublin, Ireleand), and incubated at standard conditions of 37 °C with 5% CO_2_ in a HeraCell Heraeus cell incubator (Hanau, Germany).

For the assays, cells were cultured overnight, followed by the removal and replacement of the media with fresh media containing xanthones at the previously determined GI10 concentration for 24 h. Hyperthermia treatment involved placing the cells in a Panasonic MCO-19AIC incubator (Kadoma, Japan) set at 39 °C and 5% CO_2_ for one hour immediately after adding pre-warmed media containing xanthones. The determination of GI10 values is detailed in our prior publication [9].

### 3.3. RNA Extraction and Real-Time RT PCR

Total RNA extraction and purification were performed using the GeneMATRIX Universal DNA/RNA/Protein Purification Kit (EURX, Gdańsk, Poland) following the manufacturer’s instructions. The concentration, quality, and nucleic acid purity ratio (260/280 nm) were determined spectrophotometrically using a BioPhotometer (Eppendorf, Hamburg, Germany). Further evaluation of extract purity and quality was conducted by visualizing bands on 2% agarose gels under UV light.

To assess changes in cellular stress-related mRNA, the RT^2^ Profiler™ PCR Array Human Cellular Stress Responses (GeneGlobe ID: PAHS-019Z, Qiagen, Hilden, Germany) was used, along with the RT^2^ First Strand Kit (Qiagen, Hilden, Germany) and RT^2^ SYBR Green ROX qPCR Mastermix (Qiagen, Hilden, Germany) for first-strand synthesis (RT—reverse transcription step) and real-time PCR, respectively. All procedures were conducted according to the manufacturer’s protocols. All genes that do not reach the threshold before cycle no. 35 were determined as below the detection limit and discarded. Data analysis was carried out using the RT^2^ Profiler PCR Data Analysis software available at https://geneglobe.qiagen.com/pl (accession date 6 September 2024).

### 3.4. Gene Ontology and Gene Set Enrichment Analysis

GO and gene set enrichment analysis were performed with the FunRich program v3.1.4 (accession date 30 September 2024). For gene set enrichment, genes were divided by the drug or drug + hyperthermia condition. Additional genes not investigated in our study were also included. GO genes were divided by the drug or drug + hyperthermia condition and up- or down-regulation. Only molecular functions with *p* < 0.001 were displayed.

Pathway analysis was performed with STRING database (accession date 30 September 2024). Investigated genes were divided by the drug or drug + hyperthermia condition. Genes were divided into clusters by incorporated function of MCL clustering with an infiltration parameter of 3. The edges between clusters are in dotted lines.

## 4. Conclusions

In this study, the influence of hyperthermia in combination with aminopropyl morpholine xanthone derivatives, **MAG** and **CIS** on TOV-21G and SK-OV-3 ovarian cancer cells was investigated. qPRC arrays focused on cellular stress-related genes demonstrated significant cell-specific changes in expression of ROS-related, cytochrome P450 and HSP genes. Evaluation of data indicated the contribution of ROS activity in treated cells. This study also confirms synergistic effects of hyperthermia and **CIS** especially in ROS production. This effect may be further enhanced by increasing both the temperature and concentration of the drug. In silico studies pointed to HSPA1A protein as the central node of changes induced after the treatment. Also, drug detoxification and chaperoning proteins were devised as generally influenced by these treatments. Additional studies on protein level are necessary to further elucidate the exact anticancer mechanism of xanthones and hyperthermia.

## Figures and Tables

**Table 1:** Structure and anticancer activity of tested compounds presented with their GI10 (concentration of selected drug that inhibits cell growth by 10%) [9].

		IC10 TOV-21G	IC10 SK-OV-3
**C7**	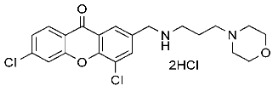	7.81 μM	11.87 μM
**C8**	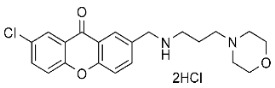	7.81 μM	31.78 μM
α-mangostin	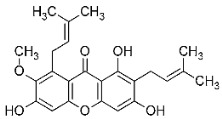	2.25 μM	40.02 μM
cisplatin	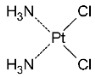	22.49 μM	2.82 μM

**C7**—4,6-dichloro-2-(((3-morpholinopropyl)amino)methyl)-9*H*-xanthen-9-one dihydrochloride; **C8**—7-chloro-2-(((3-morpholinopropyl)amino)methyl)-9*H*-xanthen-9-one dihydrochloride. Full methodology with verification of the structure, purity and melting temperature of the xanthone derivatives can be found at [9].

**Table 2:** Detailed list of genes of interest, divided into groups.

**Anti-oxidant and Pro-Oxidant Enzymes**
ACADSB, CAT, GPX1, GPX2, SOD1, SOD2, SOD3, XDH
**Xenobiotic Metabolism**
**Cytochrome P450s**
CYP11A1, CYP11B2, CYP17A1, CYP1A1, CYP1A2, CYP1B1, CYP2C19, CYP2C9, CYP2D6, CYP2E1, CYP2F1, CYP3A4, CYP4A11, CYP4B1, CYP7A1, CYP7B1
**Other Xenobiotic Metabolism Genes**
CES1, EPHX2, FMO1, FMO4, FMO5, GSR, GSTA1, GSTA5 (YC2), GSTM5
**Molecular Chaperones**
**Heat Shock Proteins**
CRYAA, CRYAB, DNAJA1, DNAJA2, DNAJA3, DNAJA4, DNAJB1, DNAJB11, DNAJB12, DNAJB2, DNAJB4, DNAJB5, DNAJB6, DNAJB9, DNAJC1, DNAJC4, DNAJC5, DNAJC6, DNAJC7, DNAJC8, DNAJC9, HMOX1, HMOX2, HSPA12A, HSPA1A (HSP70-1A), HSPA1L, HSPA2, HSPA4 (HSP70), HSPA5 (GRP78), HSPA8, HSPA9, HSPB1 (HSP27), HSPB2, HSPB3, HSPD1, HSPE1, SERPINH1 (HSP47)
**Other Molecular Chaperones**
BAG1, CALR, CANX, CCT2, CCT3, CCT4, CCT5, CCT7, CCT8, CLU, HOPX, PPID, ST13, TCP1

## Data Availability

The data presented in this study are available upon reasonable request from the corresponding author.

## Supplementary Materials

The following supporting information can be downloaded at: https://www.mdpi.com/article/10.3390/cancers17172896/s1, Figure S1: The network of interaction of underexpressed genes of (a) TOV21G and (b) SK-OV-3 after simultaneous heat and C7 treatment. Overexpressed genes were ommited due to to low numer to assess the interactions; Figure S2: The network of interaction of underexpressed genes of (a) TOV21G and (b) SK-OV-3 after simultaneous heat and C8 treatment; Figure S3: The network of interaction of overpressed genes of (a) TOV21G and (b) SK-OV-3 after simultaneous heat and C8 treatment; Figure S4: The network of interaction of overexpressed genes of TOV21G after simultaneous heat and CIS treatment. Downregulated genes were ommited due to lack of significantly changed genes for SK-OV-3 and only CYP7A1 upregulated in case of TOV-21G; Figure S5: The network of interaction of underexpressed genes of TOV21G after simultaneous heat and MAG treatment. No significantly downregulated genes were noted for SK-OV-3; Figure S6: The network of interaction of overexpressed genes of SK-OV-3 after simultaneous heat and MAG treatment. Only DNAJC8 gene was significantly upregulated noted for TOV-21G; Figure S7: The molecular functions of underexpressed genes of SK-OV-3 after heat treatment. No downregulated genes were noted for TOV-21G cell line; Figure S8: The molecular functions of underexpressed genes of (a) TOV21G and (b) SK-OV-3 after simultaneous heat and C7 treatment; Figure S9: The molecular functions of underexpressed genes of (a) TOV21G and (b) SK-OV-3 after simultaneous heat and C8 treatment; Figure S10: The molecular functions of overexpressed genes of (a) TOV21G and (b) SK-OV-3 after simultaneous heat and C8 treatment; Figure S11: The molecular functions of underexpressed genes of SK-OV-3 after simultaneous heat and CIS treatment. No downregulated genes were noted for TOV-21G cell line; Figure S12: The molecular functions of overexpressed genes of TOV21G after simultaneous heat and CIS treatment. No upregulated genes were noted for SK-OV-3 cell line; Figure S13: The molecular functions of underexpressed genes of TOV21G after simultaneous heat and MAG treatment. No downregulated genes were noted for SK-OV-3 cell line; Figure S14: The molecular functions of overexpressed genes of (a) TOV21G and (b) SK-OV-3 after simultaneous heat and MAG treatment. Figure S15: STRING proteins interaction pathway analysis of (a) TOV-21G and (b) SK-OV-3 cells after simultaneous heat and C7 treatment. Proteins were grouped into clusters, based on their molecular function. Dotted lines represents the edges between clusters; Figure S16: STRING proteins interaction pathway analysis of (a) TOV-21G and (b) SK-OV-3 cells after simultaneous heat and C8 treatment. Proteins were grouped into clusters, based on their molecular function. Dotted lines represents the edges between clusters; Figure S17: STRING proteins interaction pathway analysis of (a) TOV-21G and (b) SK-OV-3 cells after simultaneous heat and MAG treatment. Proteins were grouped into clusters, based on their molecular function. Dotted lines represents the edges between clusters; Figure S18: STRING proteins interaction pathway analysis of TOV-21G cells after simultaneous heat and CIS treatment. Only CYP7A1 was downregulated in SK-OV-3 cell line. Proteins were grouped into clusters, based on their molecular function. Dotted lines represents the edges between clusters.
