# Supplementary material for: Synergistic Effects of Novel Xanthone Derivatives and Mild Hyperthermia in Ovarian Cancer: Insights from Gene Expression and In Silico Analyses
Source: Cancers (Basel). 2025 Sep 3;17(17):2896. doi: 10.3390/cancers17172896 (PMC12427508; doi:10.3390/cancers17172896)
Supplement: Supplementary file 1 [file cancers-17-02896-s001.zip › Supplementary Materials S1.pptx]

## Slide 1
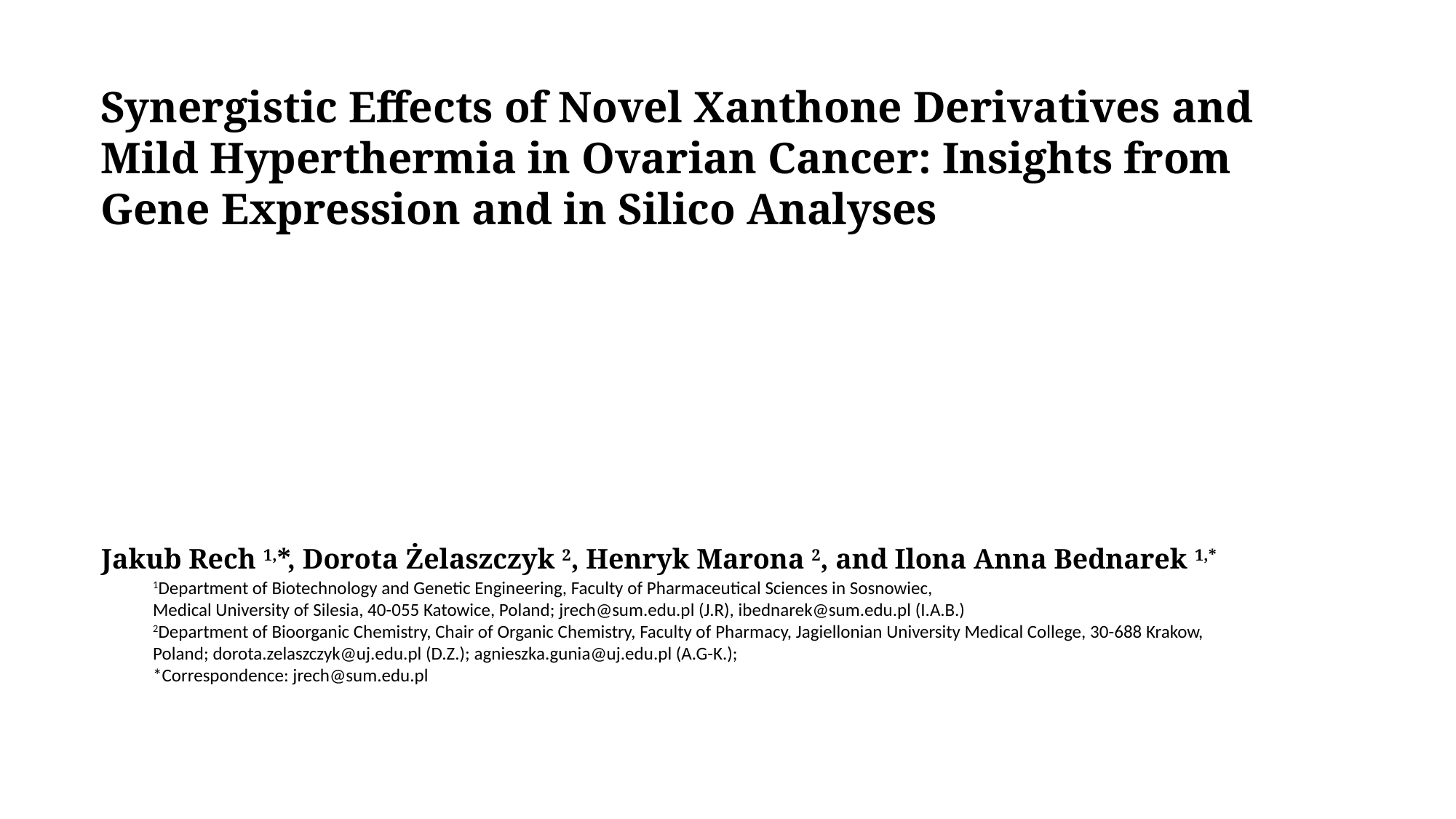

Synergistic Effects of Novel Xanthone Derivatives and Mild Hyperthermia in Ovarian Cancer: Insights from Gene Expression and in Silico Analyses
# Jakub Rech 1,*, Dorota Żelaszczyk 2, Henryk Marona 2, and Ilona Anna Bednarek 1,*
1Department of Biotechnology and Genetic Engineering, Faculty of Pharmaceutical Sciences in Sosnowiec,
Medical University of Silesia, 40-055 Katowice, Poland; jrech@sum.edu.pl (J.R), ibednarek@sum.edu.pl (I.A.B.)
2Department of Bioorganic Chemistry, Chair of Organic Chemistry, Faculty of Pharmacy, Jagiellonian University Medical College, 30-688 Krakow, Poland; dorota.zelaszczyk@uj.edu.pl (D.Z.); agnieszka.gunia@uj.edu.pl (A.G-K.);
*Correspondence: jrech@sum.edu.pl

## Slide 2
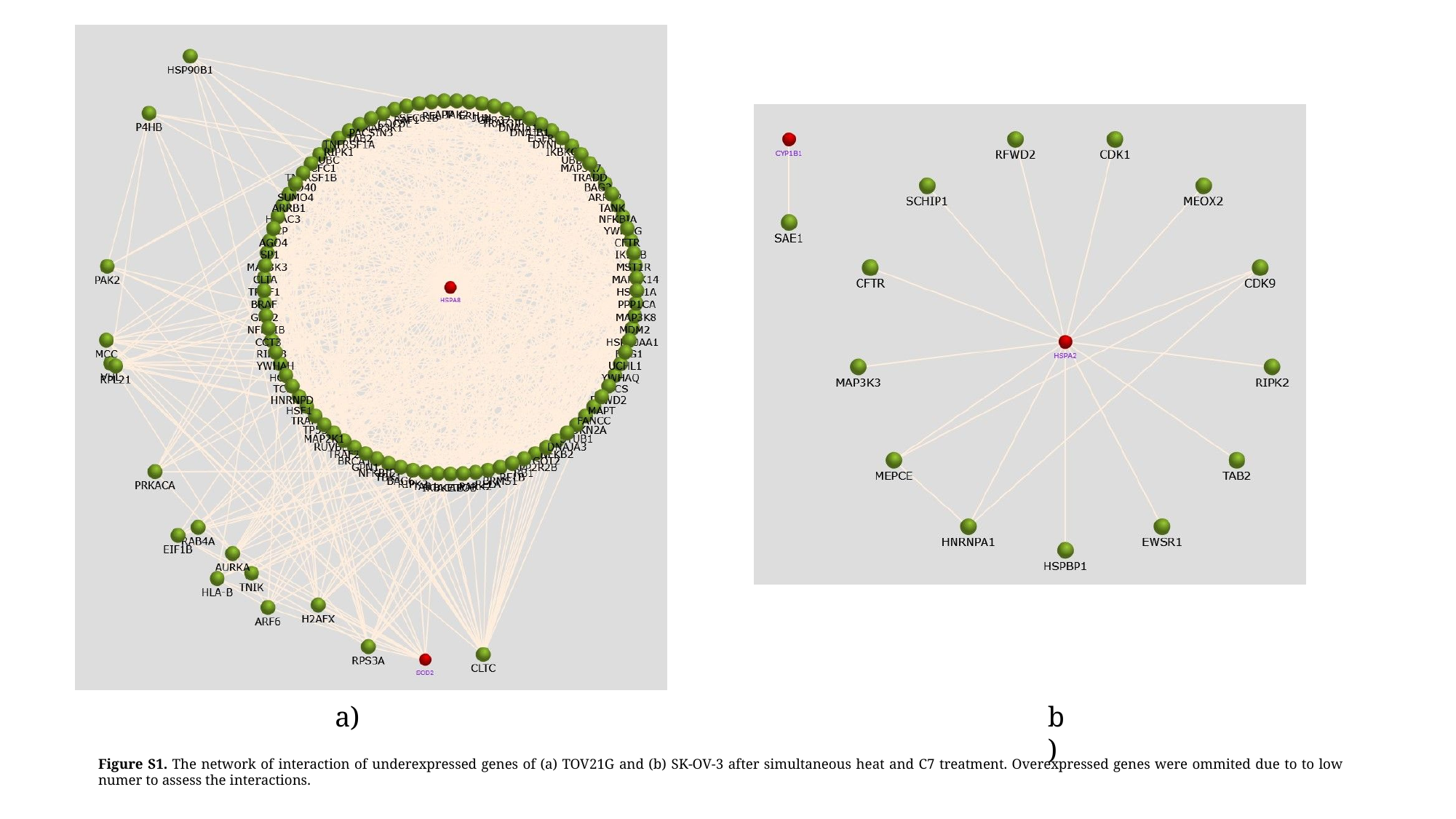

a)
b)
Figure S1. The network of interaction of underexpressed genes of (a) TOV21G and (b) SK-OV-3 after simultaneous heat and C7 treatment. Overexpressed genes were ommited due to to low numer to assess the interactions.

## Slide 3
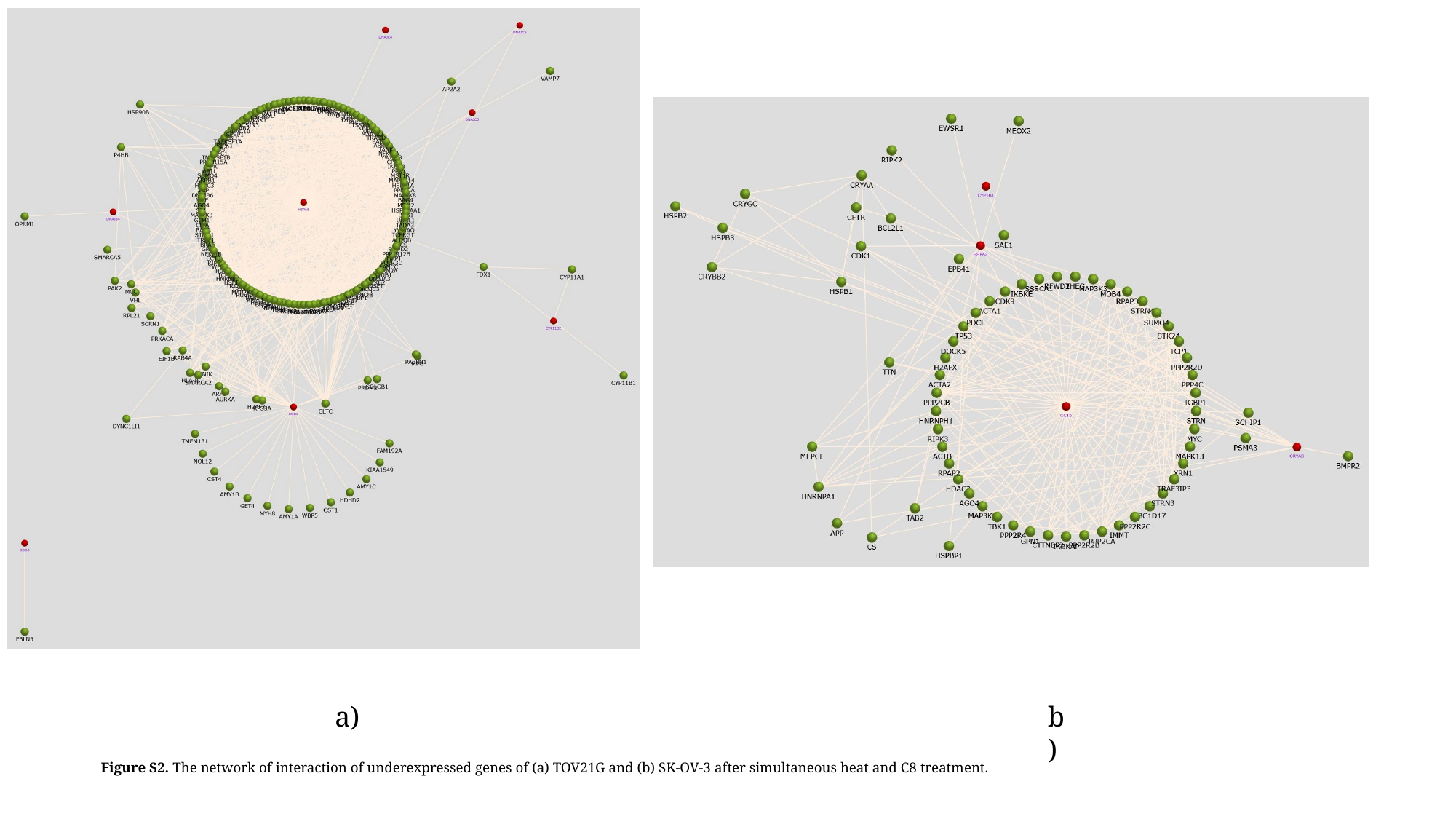

#
a)
b)
Figure S2. The network of interaction of underexpressed genes of (a) TOV21G and (b) SK-OV-3 after simultaneous heat and C8 treatment.

## Slide 4
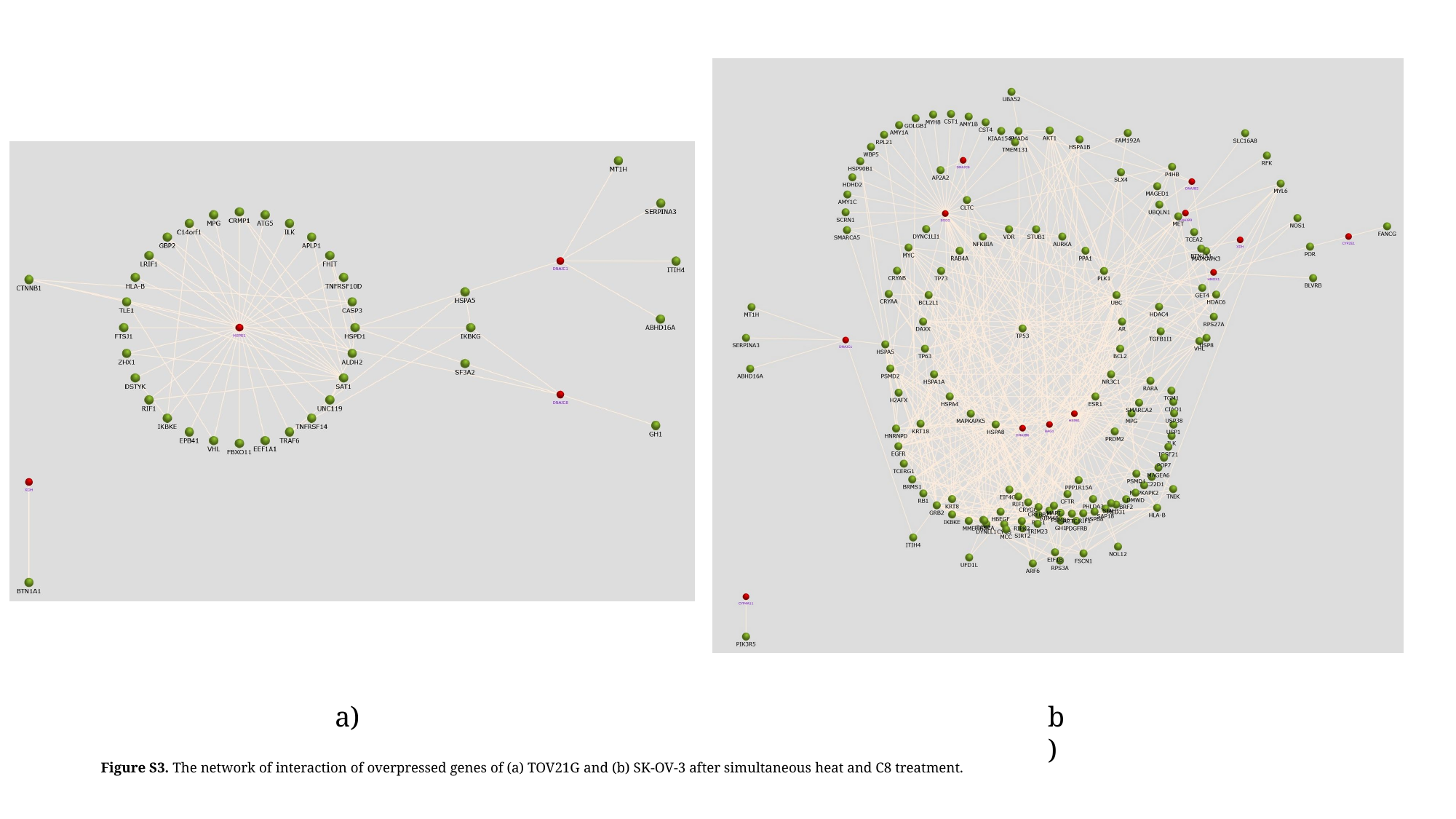

#
a)
b)
Figure S3. The network of interaction of overpressed genes of (a) TOV21G and (b) SK-OV-3 after simultaneous heat and C8 treatment.

## Slide 5
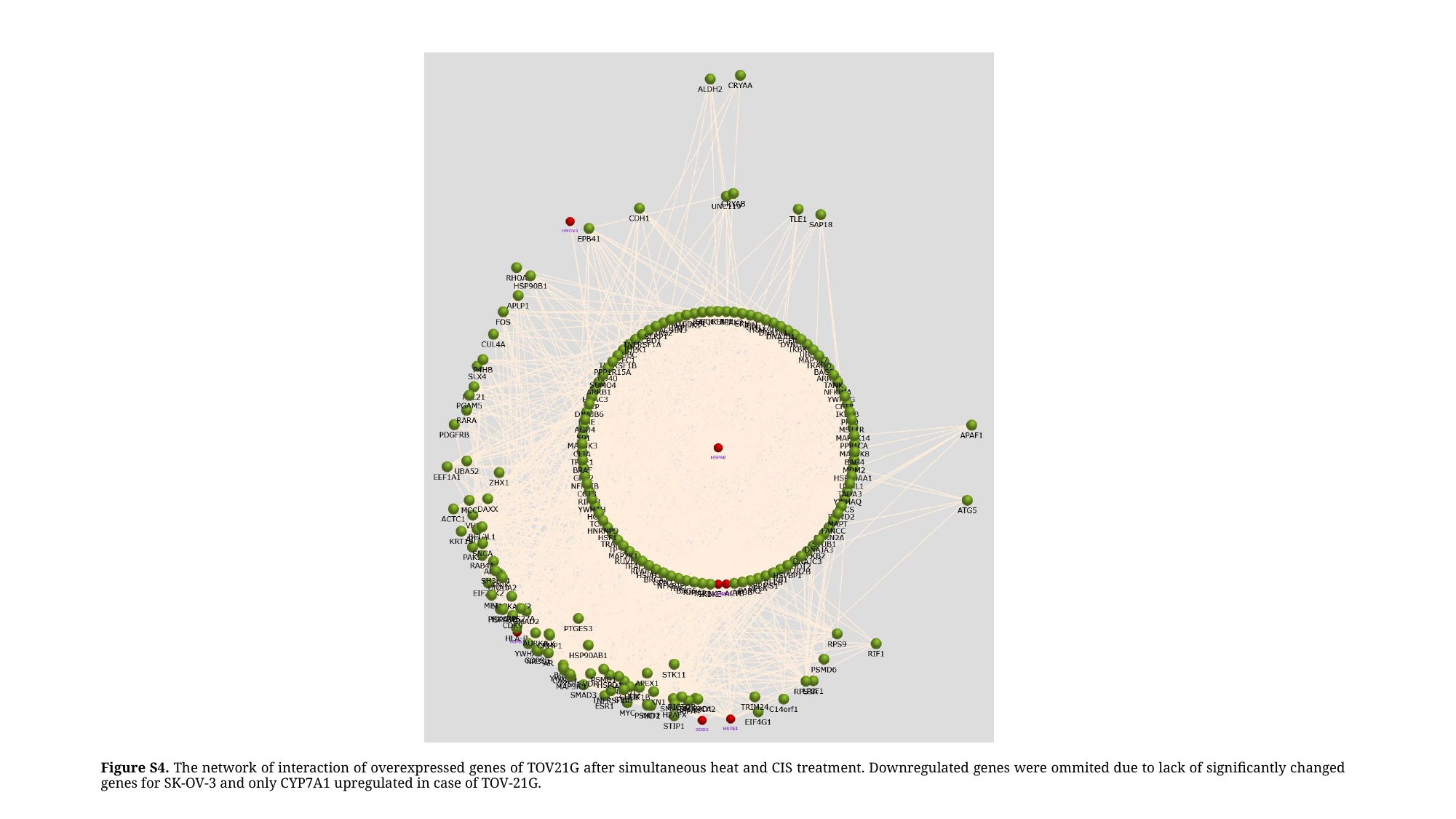

#
Figure S4. The network of interaction of overexpressed genes of TOV21G after simultaneous heat and CIS treatment. Downregulated genes were ommited due to lack of significantly changed genes for SK-OV-3 and only CYP7A1 upregulated in case of TOV-21G.

## Slide 6
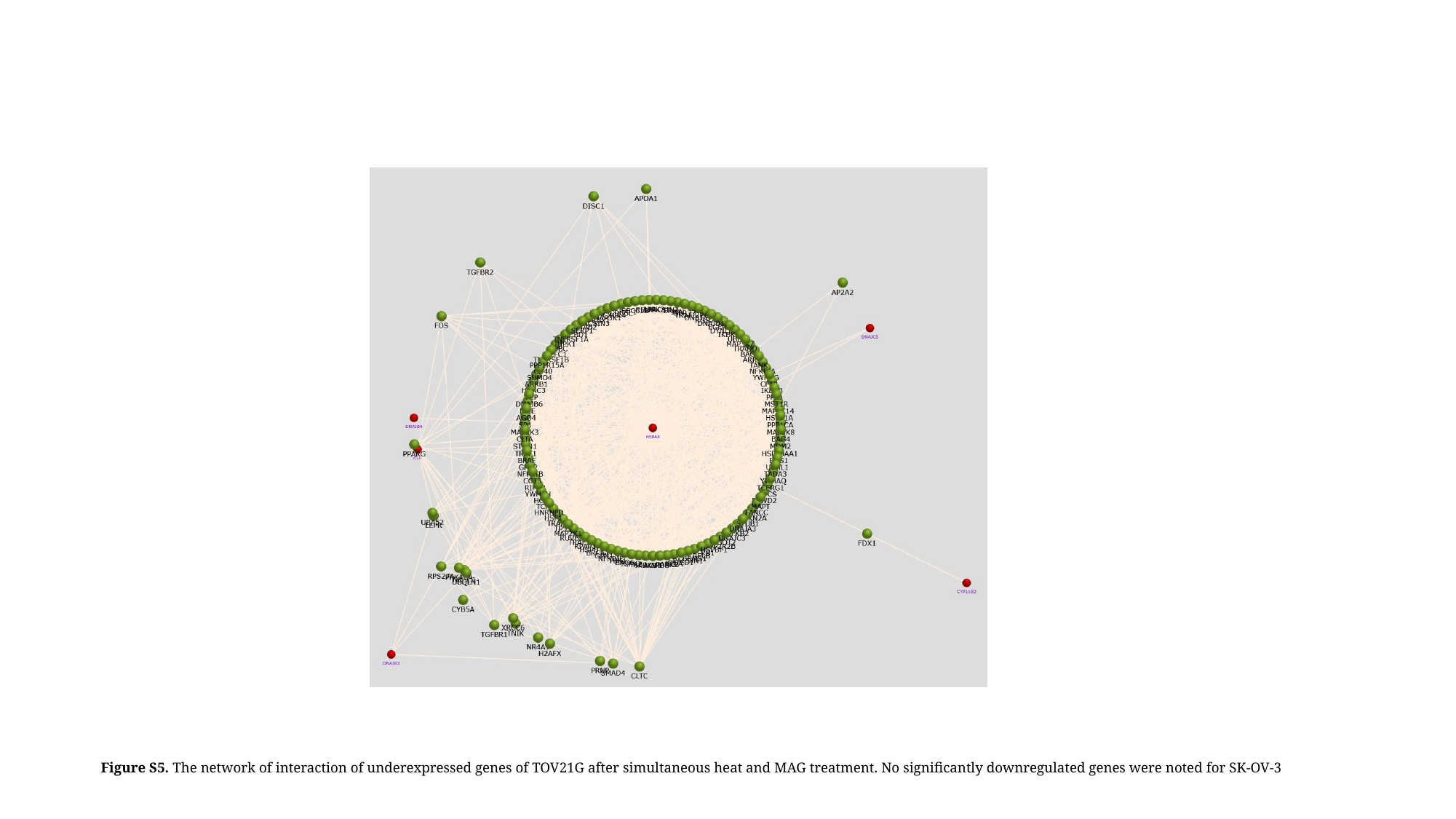

#
Figure S5. The network of interaction of underexpressed genes of TOV21G after simultaneous heat and MAG treatment. No significantly downregulated genes were noted for SK-OV-3

## Slide 7
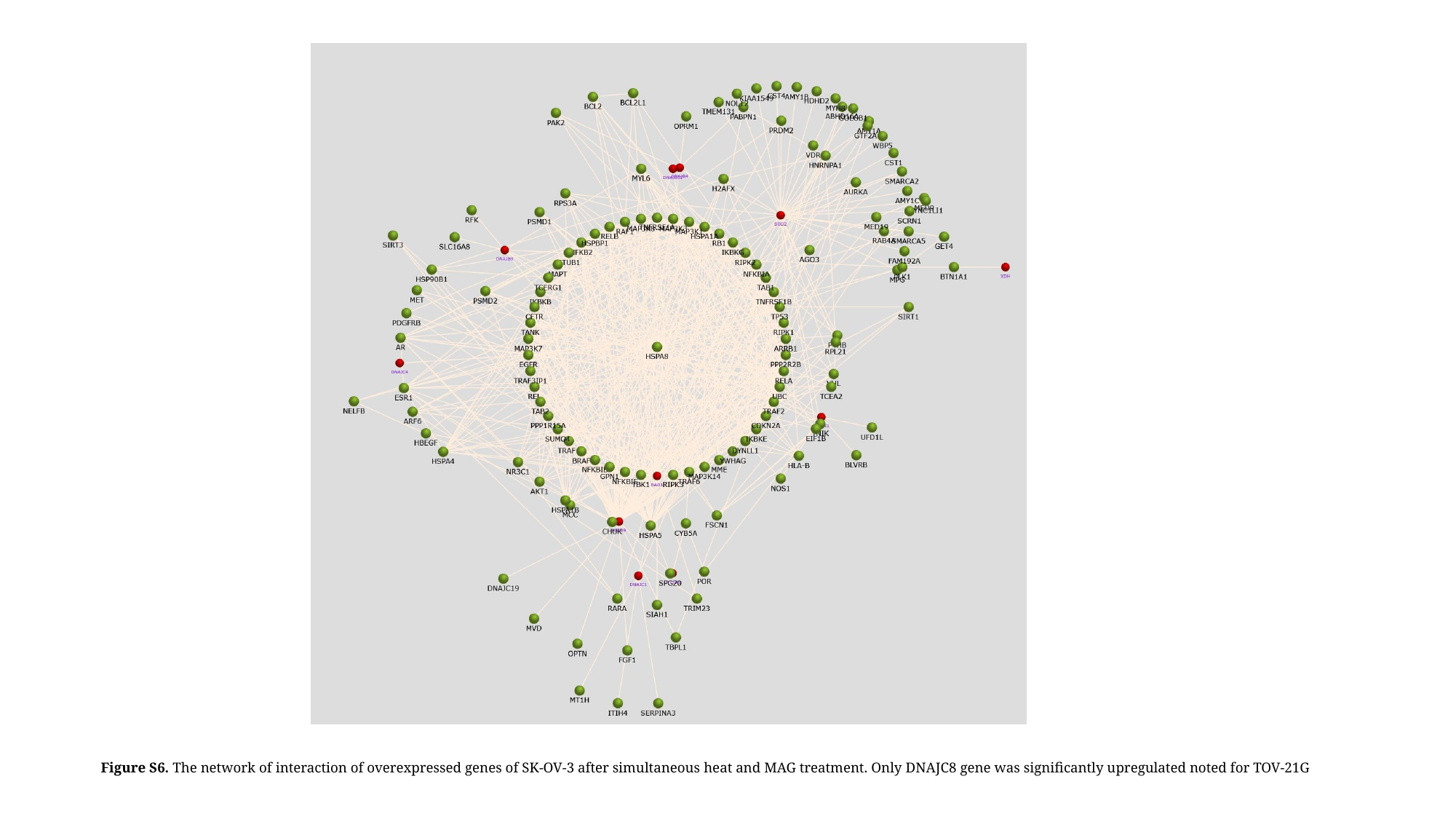

#
Figure S6. The network of interaction of overexpressed genes of SK-OV-3 after simultaneous heat and MAG treatment. Only DNAJC8 gene was significantly upregulated noted for TOV-21G

## Slide 8
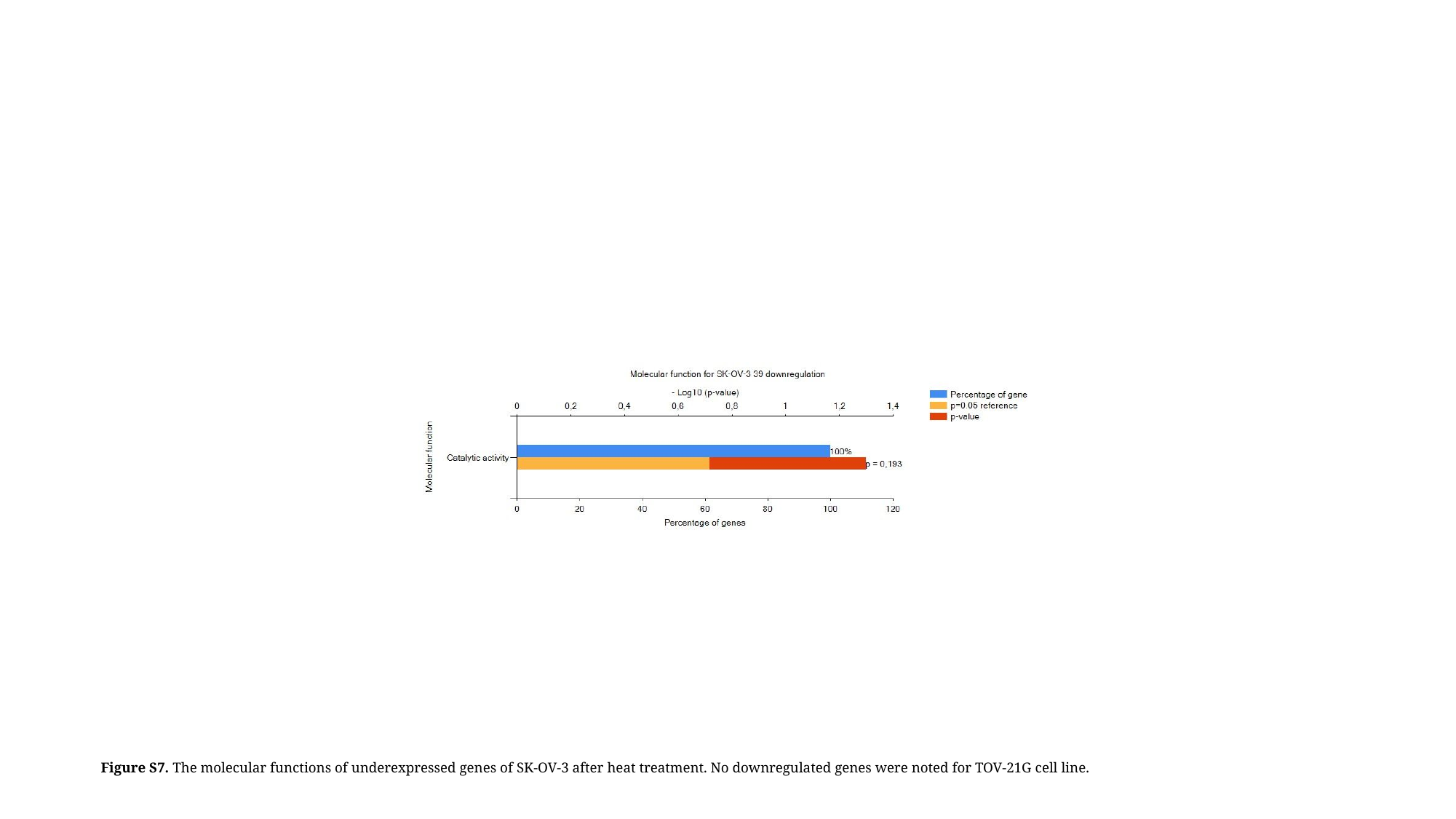

#
Figure S7. The molecular functions of underexpressed genes of SK-OV-3 after heat treatment. No downregulated genes were noted for TOV-21G cell line.

## Slide 9
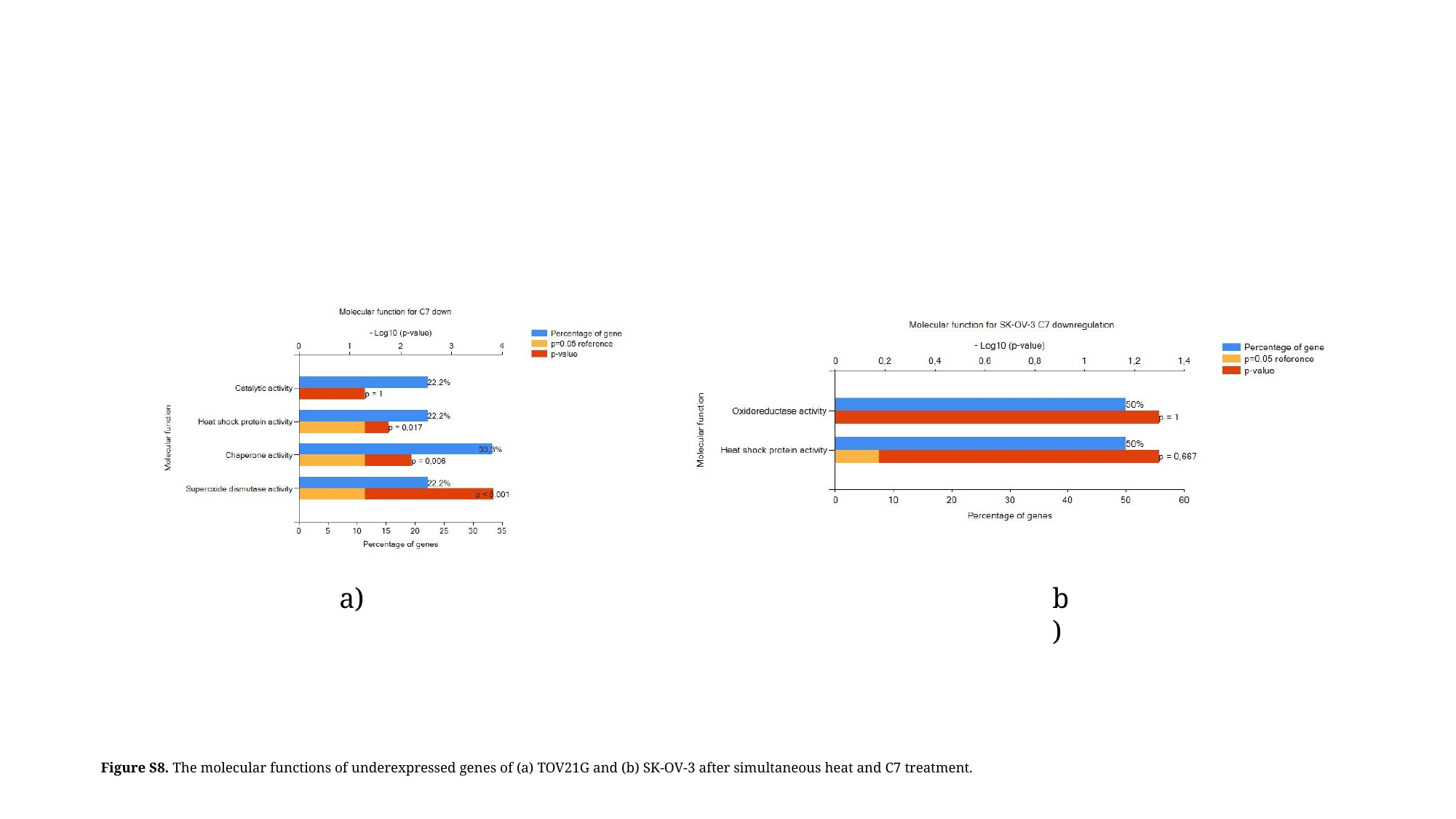

#
a)
b)
Figure S8. The molecular functions of underexpressed genes of (a) TOV21G and (b) SK-OV-3 after simultaneous heat and C7 treatment.

## Slide 10
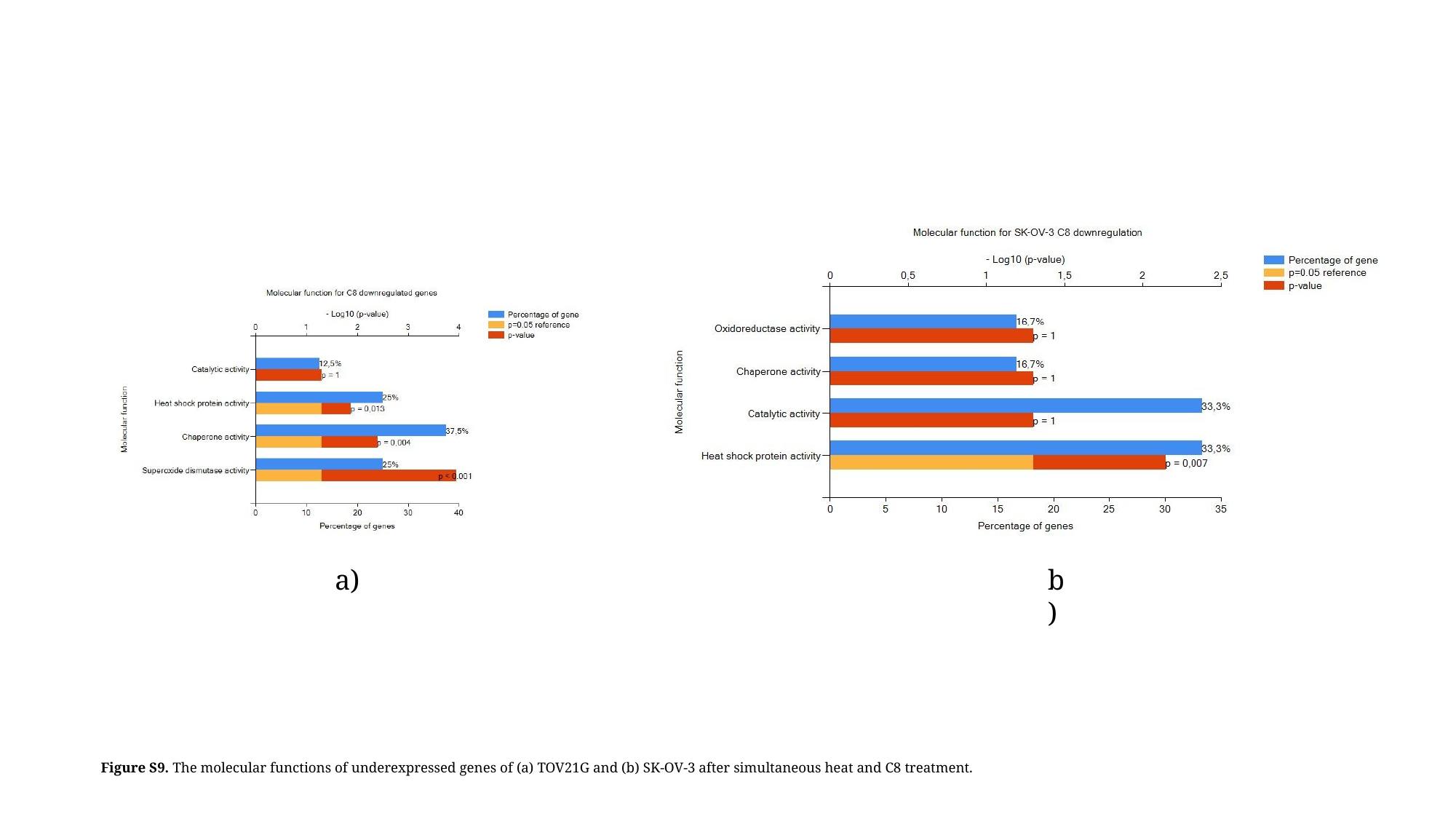

#
a)
b)
Figure S9. The molecular functions of underexpressed genes of (a) TOV21G and (b) SK-OV-3 after simultaneous heat and C8 treatment.

## Slide 11
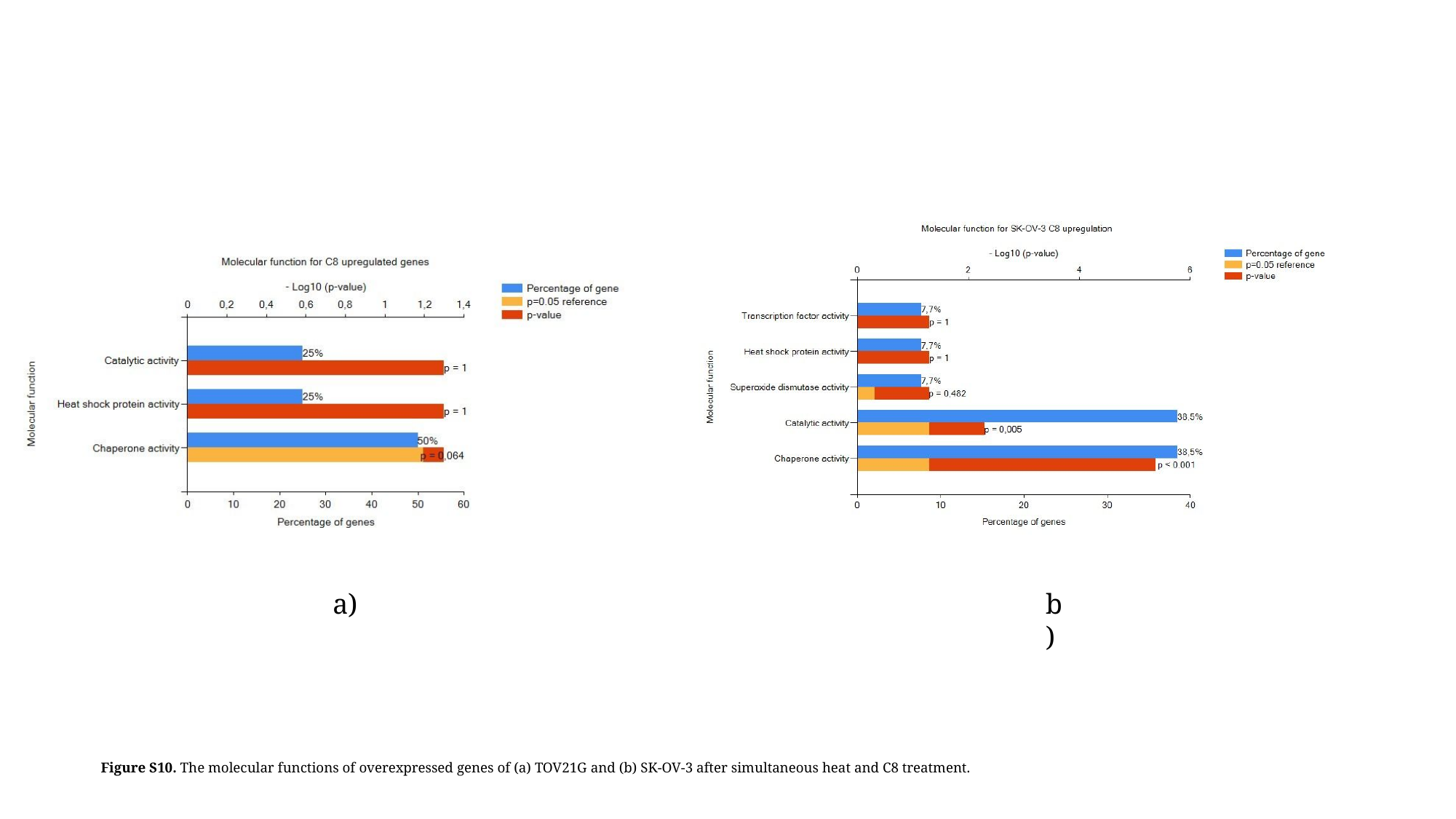

#
a)
b)
Figure S10. The molecular functions of overexpressed genes of (a) TOV21G and (b) SK-OV-3 after simultaneous heat and C8 treatment.

## Slide 12
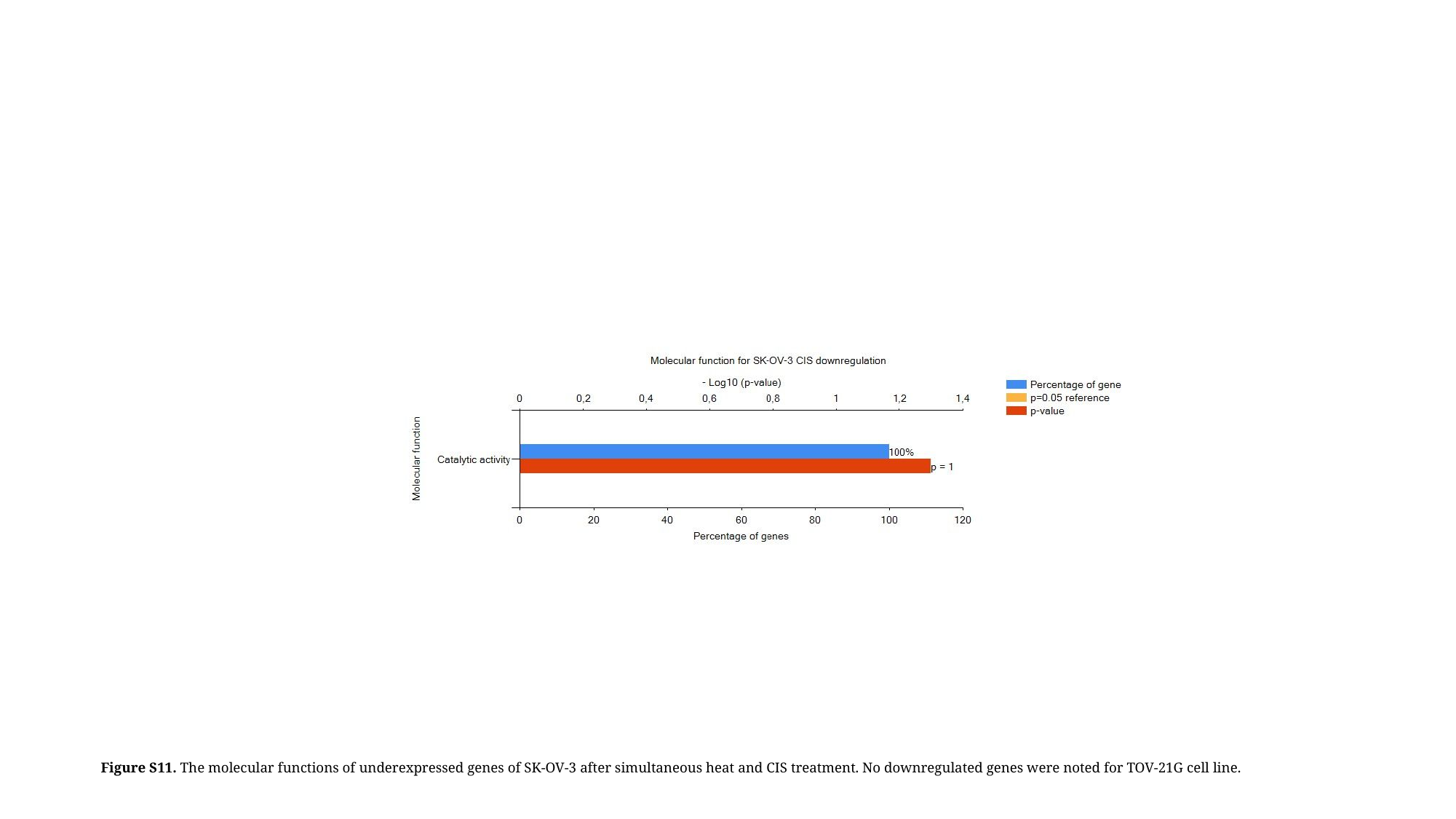

#
Figure S11. The molecular functions of underexpressed genes of SK-OV-3 after simultaneous heat and CIS treatment. No downregulated genes were noted for TOV-21G cell line.

## Slide 13
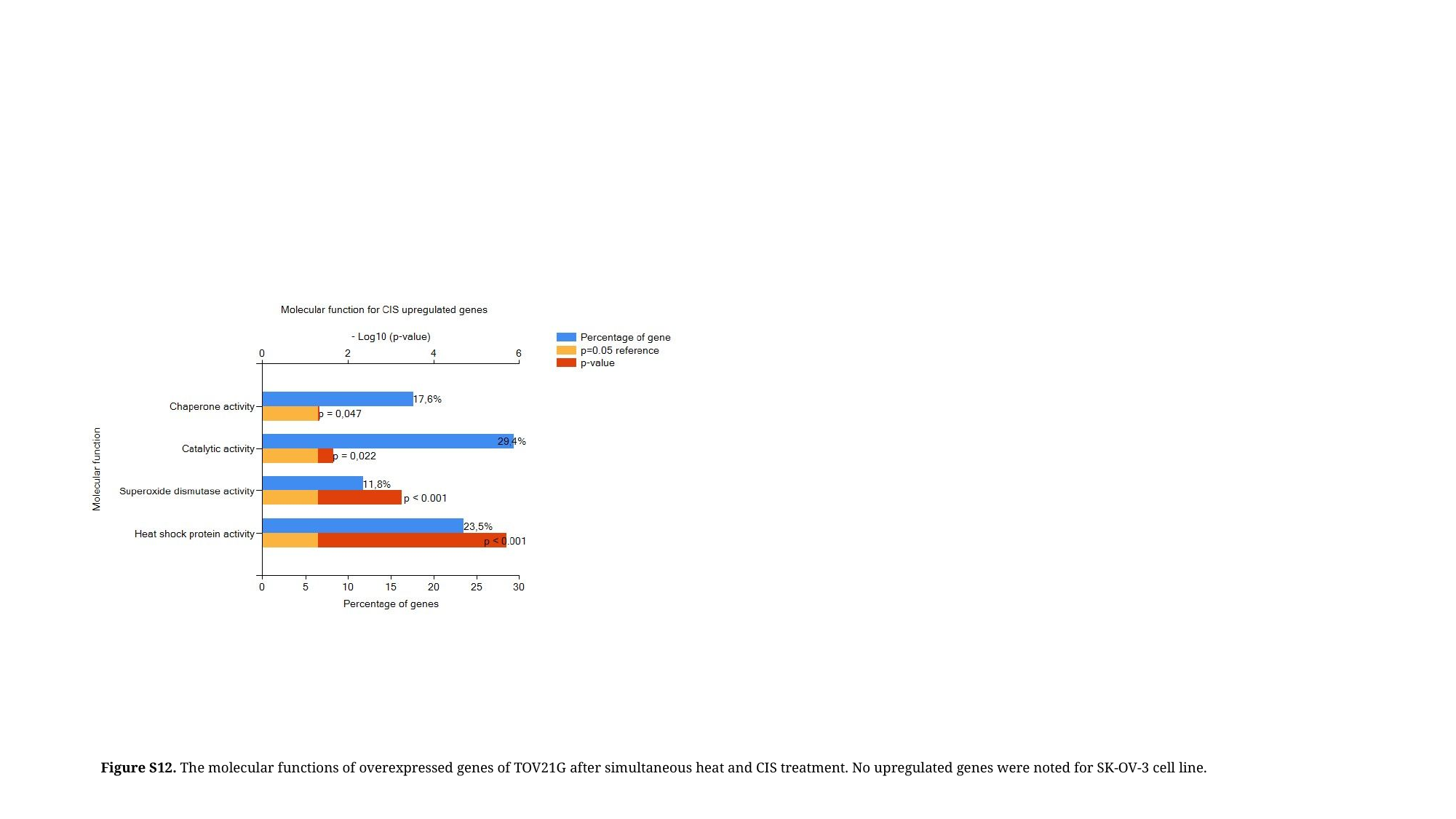

#
Figure S12. The molecular functions of overexpressed genes of TOV21G after simultaneous heat and CIS treatment. No upregulated genes were noted for SK-OV-3 cell line.

## Slide 14
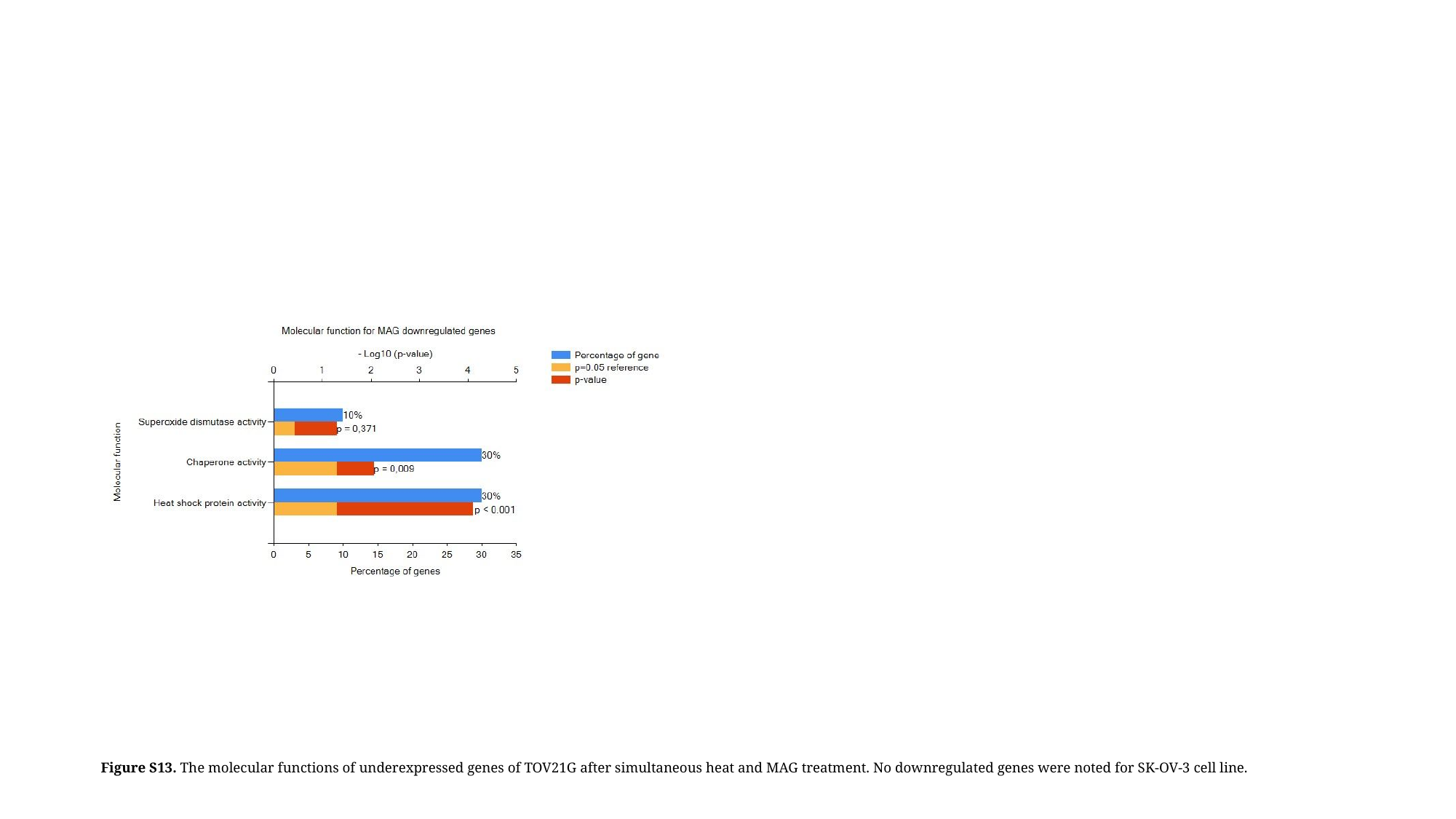

#
Figure S13. The molecular functions of underexpressed genes of TOV21G after simultaneous heat and MAG treatment. No downregulated genes were noted for SK-OV-3 cell line.

## Slide 15
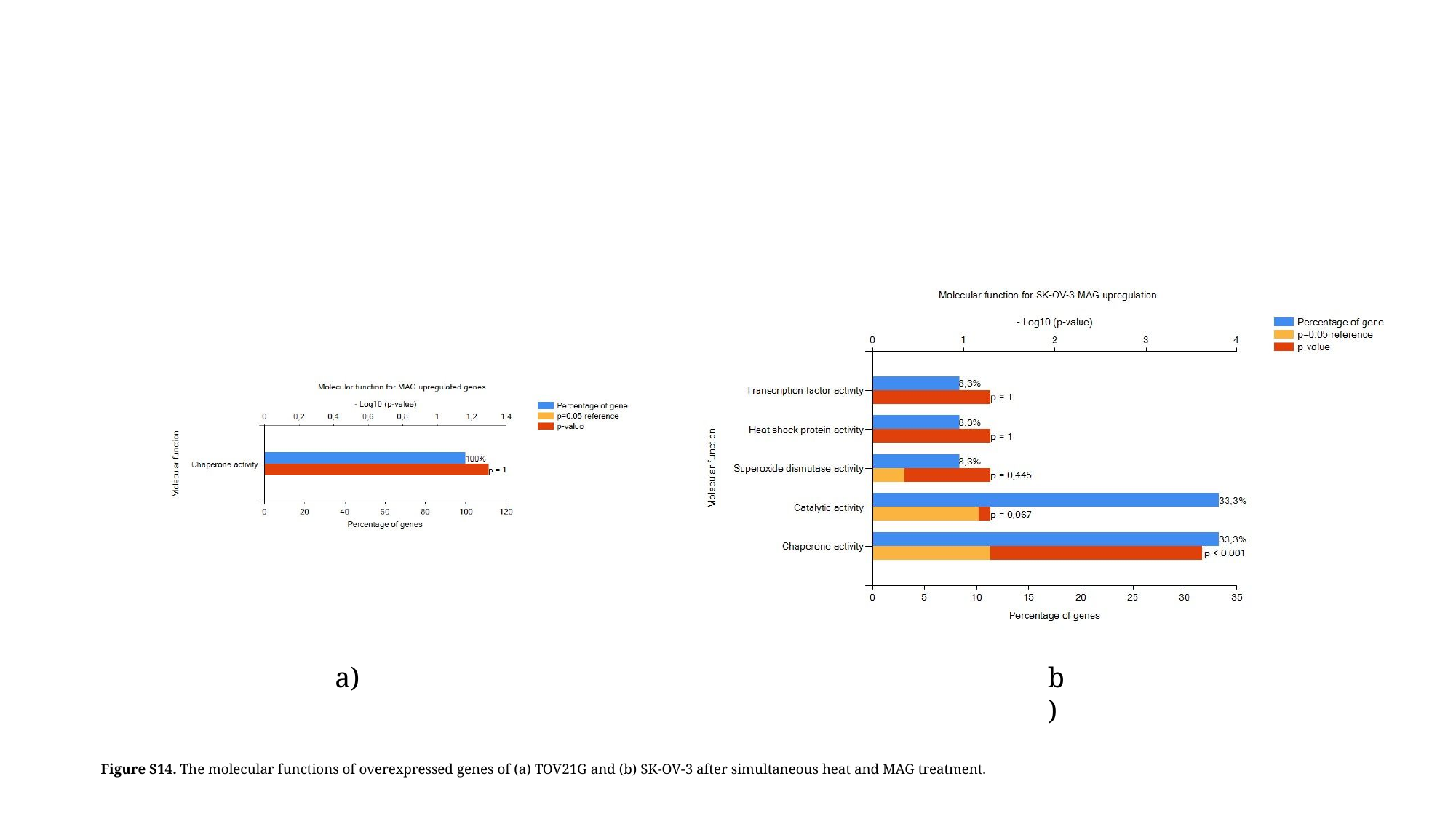

#
a)
b)
Figure S14. The molecular functions of overexpressed genes of (a) TOV21G and (b) SK-OV-3 after simultaneous heat and MAG treatment.

## Slide 16
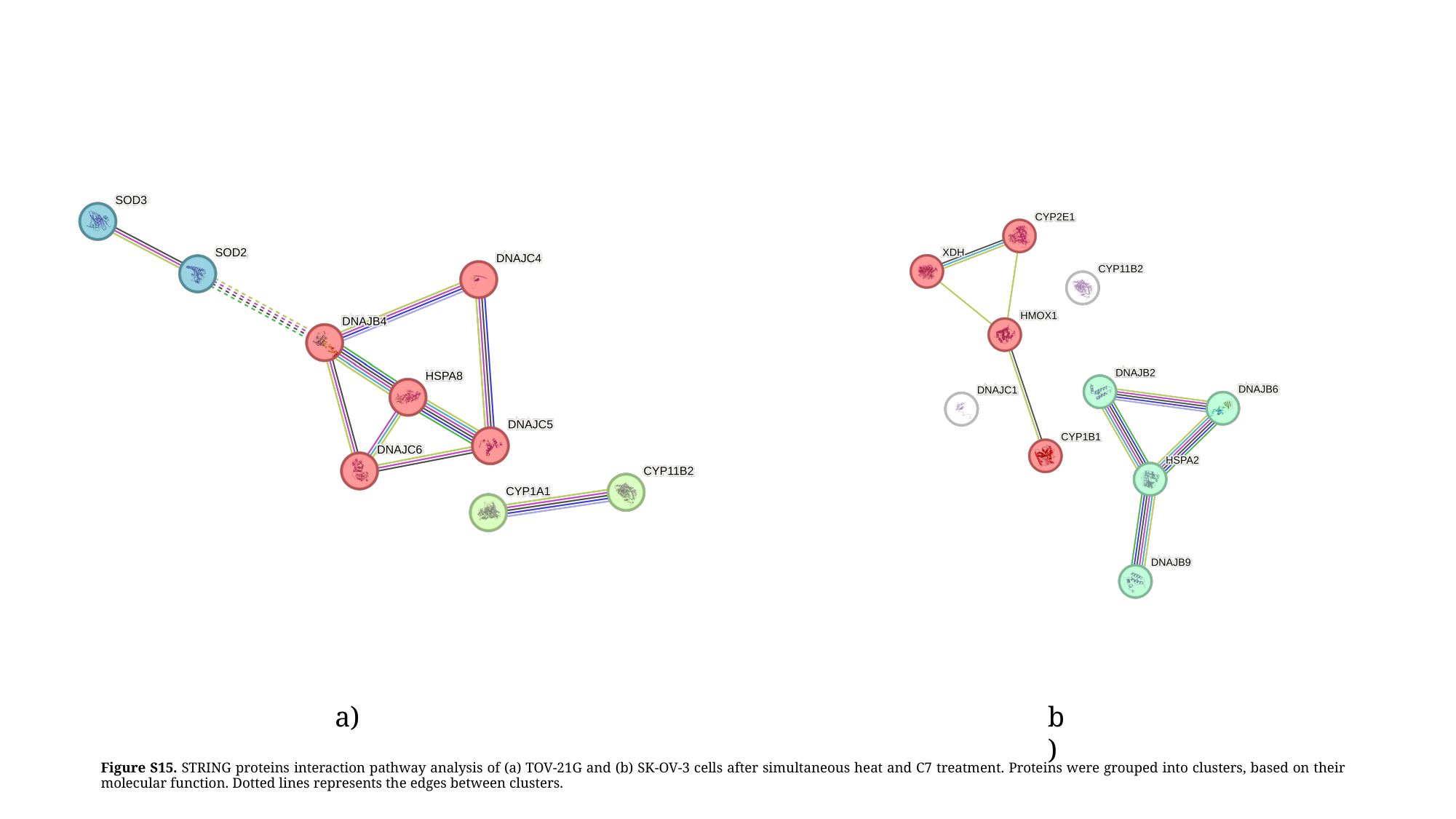

#
a)
b)
Figure S15. STRING proteins interaction pathway analysis of (a) TOV-21G and (b) SK-OV-3 cells after simultaneous heat and C7 treatment. Proteins were grouped into clusters, based on their molecular function. Dotted lines represents the edges between clusters.

## Slide 17
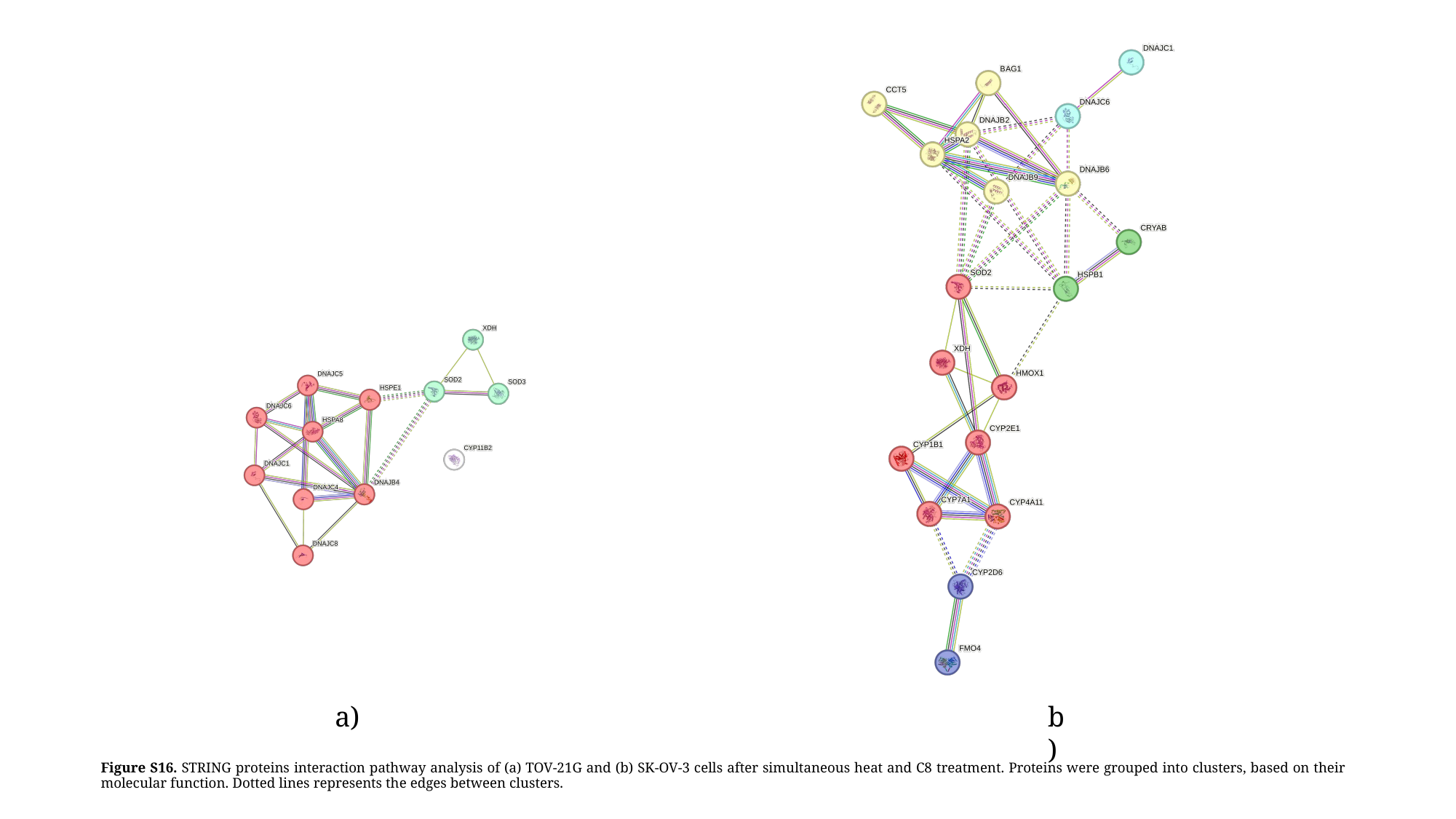

#
a)
b)
Figure S16. STRING proteins interaction pathway analysis of (a) TOV-21G and (b) SK-OV-3 cells after simultaneous heat and C8 treatment. Proteins were grouped into clusters, based on their molecular function. Dotted lines represents the edges between clusters.

## Slide 18
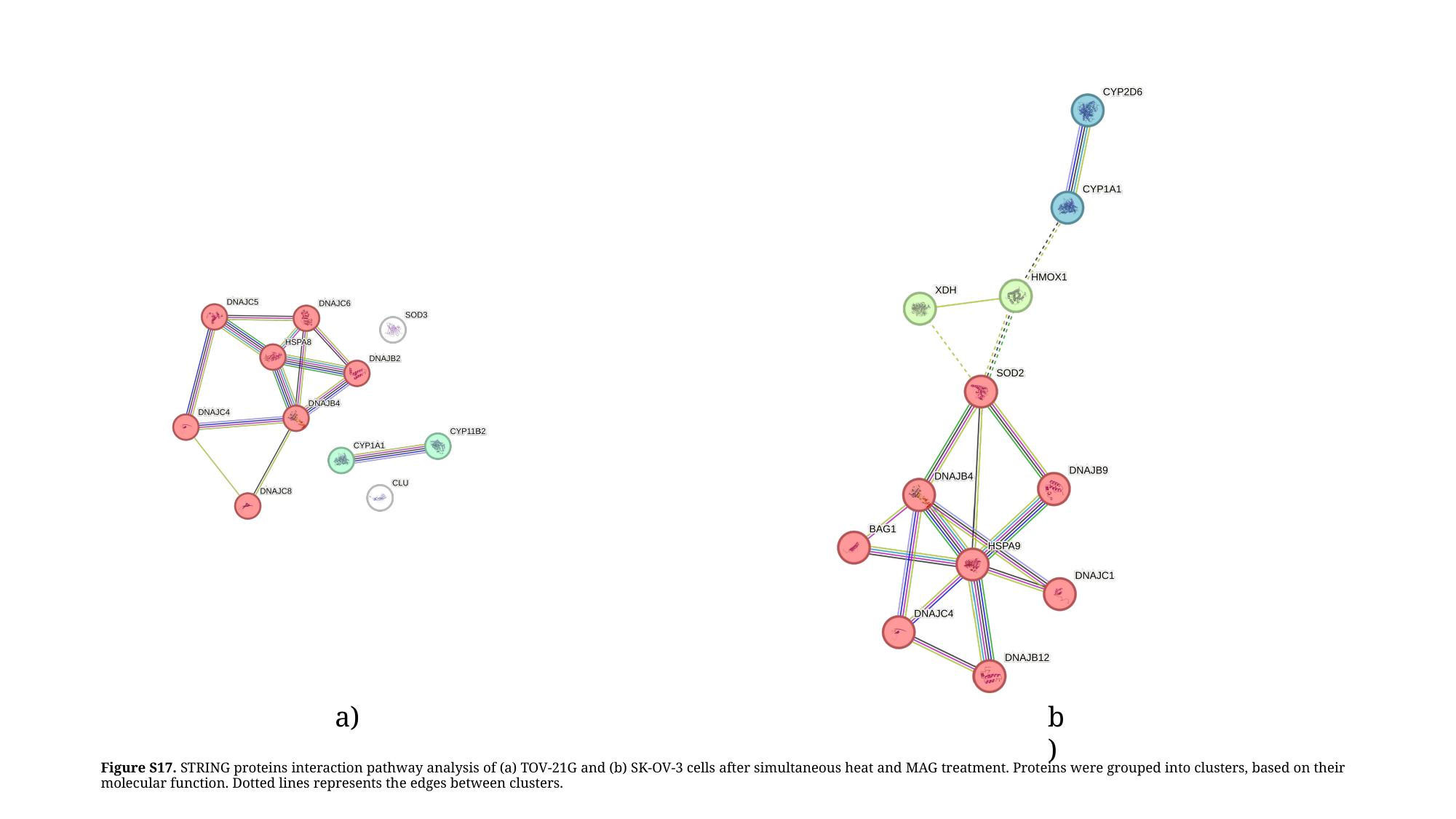

#
a)
b)
Figure S17. STRING proteins interaction pathway analysis of (a) TOV-21G and (b) SK-OV-3 cells after simultaneous heat and MAG treatment. Proteins were grouped into clusters, based on their molecular function. Dotted lines represents the edges between clusters.

## Slide 19
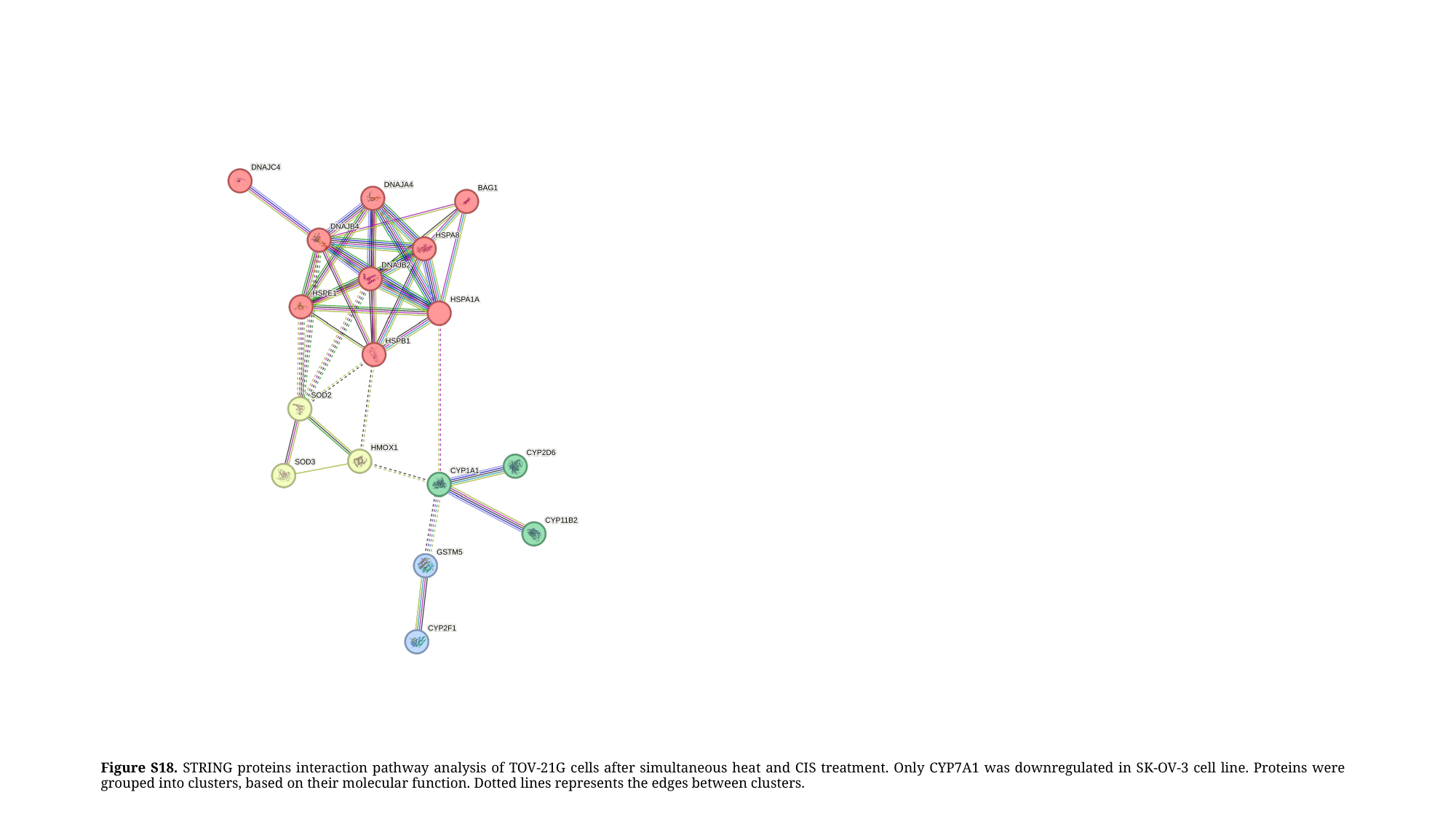

#
Figure S18. STRING proteins interaction pathway analysis of TOV-21G cells after simultaneous heat and CIS treatment. Only CYP7A1 was downregulated in SK-OV-3 cell line. Proteins were grouped into clusters, based on their molecular function. Dotted lines represents the edges between clusters.
